# Efficacy and safety of transcutaneous electrical acupoint stimulation and acupressure in alleviating chemotherapy-related adverse reactions in female patients with breast cancer: a randomized clinical trial

**DOI:** 10.3389/fonc.2026.1788635

**Published:** 2026-04-15

**Authors:** Jiang Yuan, Jie Chu, Xiuying Hu, Xinyu Zhao, Hao Li, Binwen Zhang, Li Baihong, Jinshu Han, Zhizhen Tao, Tumalisi Kayishaer, Chao Tian, Bingmei Zhu, Yi Yang

**Affiliations:** 1 School of Public Health, Chengdu University of Traditional Chinese Medicine, Cheng Du, China; 2 School of Medical, University of Electronic Science and Technology of China, Cheng Du, China; 3 Innovation Center of Nursing Research, Nursing Key Laboratory of Sichuan Province, West China Hospital, Sichuan University, Chengdu, Sichuan, China; 4 School of Management, Chengdu University of Traditional Chinese Medicine, Cheng Du, China; 5 Division of internal Medicine, Institute of Integrated Traditional Chinese and Western Medicine, Regenerative Medicine Research Center, West China Hospital, Sichuan University, Cheng Du, China; 6 Dept of Disciplinary Development, Sichuan University, West China School of Medicine, West China Hosp, Cheng Du, China; 7 School of Clinical Medicine, Chengdu University of Traditional Chinese Medicine, Cheng Du, China; 8 Department of Breast Surgery, Sichuan Clinical Research Center for Cancer, Sichuan Cancer Hospital and Institute, Sichuan Cancer Center, University of Electronic Science and Technology of China, Chengdu, China

**Keywords:** transcutaneous electrical acupoint stimulation, acupressure, breast cancer, nausea and vomiting, sleep disorders, anxiety and depression, quality of life

## Abstract

**Background:**

Common adverse reactions to breast cancer chemotherapy (nausea, vomiting, sleep disorders, anxiety, depression) are often suboptimally managed. These symptoms undermine patients’s quality of life and reduce chemotherapy adherence. Two non-invasive Traditional Chinese Medicine (TCM) interventions—transcutaneous electrical acupoint stimulation (TEAS) and acupressure have demonstrated efficacy in alleviating such discomforts, data on their effectiveness and dynamic therapeutic characteristics throughout the full chemotherapy course remain insufficient.

**Method:**

198 breast cancer patients receiving chemotherapy were subjected to simple randomization using R4.4.2, and randomly assigned to the control group, TEAS group, and acupressure group for a longitudinal follow-up over four chemotherapy cycles. Generalized estimating equations (GEE) were used to analyze intervention effects and *post-hoc* multiple comparisons, supplemented by intention-to-treat (ITT) analysis and per-protocol set (PPS) analysis.

**Results:**

Main time effect: With the progression of chemotherapy cycles, the risk of delayed chemotherapy-induced nausea and vomiting (delayed CINV) significantly increased; patients’ anxiety levels were higher in the early stage of chemotherapy cycles and showed a downward trend after the 3rd cycle. Main intervention effect: TEAS significantly improved patients’ quality of life(QOL), while acupressure primarily alleviated psychological symptoms such as anxiety and depression. Interaction effect: The therapeutic effect of TEAS was cumulative, exerting more pronounced benefits in reducing the risk of delayed CINV, improving sleep quality, and relieving delayed nausea during the mid-to-late stages of chemotherapy (cycles 3-4); acupressure achieved optimal anxiety relief in the early stage (cycle 1) and progressively alleviated delayed nausea and enhanced QOL throughout the treatment course.

**Conclusion:**

TEAS and acupressure exhibit dynamic and distinct effects in mitigating adverse reactions in breast cancer patients undergoing chemotherapy. TEAS is more suitable for the comprehensive improvement of physical symptoms and QOL during the mid-to-late stages of chemotherapy, while acupressure is preferred for early anxiety relief and gradual improvement of psychosomatic symptoms throughout the treatment. These findings provide a reference for the development of individualized non-invasive clinical intervention strategies.

**Clinical trial registration:**

https://www.chictr.org.cn/, identifier ChiCTR2300077667.

## 1 Introduction

Breast cancer is the most common malignant tumor among women worldwide (1). As a core component of the comprehensive treatment regimen, chemotherapy extends patients’ survival while being accompanied by various severe adverse reactions. Among these, chemotherapy-induced nausea and vomiting (CINV), anxiety, depression, and sleep disturbances constitute three core issues affecting patients’ treatment outcomes and prognosis (2–4). These three conditions are not mutually exclusive: persistent distress caused by CINV can directly induce or exacerbate anxiety and depression, while psychological stress and decreased sleep quality further lower the body’s tolerance threshold to chemotherapy-related adverse reactions, forming a vicious cycle of “nausea and vomiting - sleep disturbances - anxiety and depression”. Ultimately, this leads to reduced treatment adherence and deterioration of health-related quality of life (HRQOL), which refers to physical, functional, emotional, and social well-being specifically affected by breast cancer and its treatment, distinct from generic quality of life. In this study, HRQOL was assessed using the Functional Assessment of Cancer Therapy-Breast (FACT-B) questionnaire, a disease-specific measure for patients with breast cancer. Such impairment in HRQOL may even impact long-term survival (2, 5–7). Due to their physiological hormonal characteristics and psychological sensitivity to the disease, female breast cancer patients have a significantly higher incidence of CINV compared to male breast cancer patients and those with other cancer types (8). Even with strict adherence to international guidelines for standardized antiemetic therapy, nearly half of patients still experience refractory CINV symptoms (9, 10), with more prominent psychological and sleep issues.

Given the limitations of conventional western medical interventions in the comprehensive management of chemotherapy-related symptoms such as nausea and vomiting, sleep disturbances, anxiety, and depression (10, 11) bringing in constipation, headache, and increased liver and kidney burden due to polypharmacy, non-invasive, safe, and easily scalable traditional Chinese medicine (TCM)-based interventions have garnered increasing attention (12, 13). Transcutaneous Electrical Acupoint Stimulation (TEAS), an innovative form of modern acupuncture, delivers low-frequency electrical stimulation to acupoints via electrodes. It retains the core mechanism of meridian regulation while reducing infection risk and discomfort through its non-invasive nature, thereby significantly improving patient acceptability (14, 15). Acupressure, by contrast, stimulates the circulation of qi and blood in meridians through manual pressure on specific acupoints. It offers advantages of simple operation, low cost, and home-based accessibility, making it more suitable for long-term promotion (16, 17). The significance of directly comparing the two interventions lies in: clarifying the differences in efficacy and patient tolerance resulting from the differences in intervention methods and treatment intensity between the two non-invasive interventions, and providing evidence-based guidance for clinical practice to help doctors and patients jointly select the appropriate treatment plan based on individual circumstances. Existing studies have demonstrated the efficacy of TEAS in alleviating postoperative nausea and vomiting (PONV) (18–21), but no consensus has been reached regarding its effectiveness in reducing CINV incidence among chemotherapy patients (22, 23). Although acupressure (particularly auricular acupressure) can mitigate CINV symptoms in breast cancer patients, multiple studies have indicated a significant placebo effect, casting doubt on the stability of its therapeutic outcomes (24–26). In the field of psychological and sleep interventions, acupuncture and acupressure have been proven to improve sleep quality and reduce depression in individuals with insomnia (27, 28), alleviate preoperative anxiety in surgical patients (29), and enhance sleep quality and QOL in cancer patients (30). However, for the specific context of chemotherapy, there is a paucity of research exploring the synergistic effects of these two interventions in simultaneously alleviating CINV, anxiety, depression, and sleep disturbances in breast cancer patients. Additionally, high-quality randomized controlled trials (RCTs) providing long-term evidence are scarce, and the differences in therapeutic efficacy between TEAS and acupressure remain unclear. Furthermore, there is no standard yet for the selection of acupoints as determined by research. In this study, the acupoint selection principle based on the traditional Chinese medicine theory of “treating both the symptoms and the root cause” was adopted (31). Four acupoints, namely Hegu, Neiguan, Zusanli, and Sanyinjiao, were selected. Through the methods of distant and close acupoint pairing and treatment of exterior and interior aspects together, the comprehensive therapeutic effect of improving adverse reactions was achieved.

This study adopted an RCT design combined with longitudinal follow-up, enrolling female breast cancer patients undergoing chemotherapy to test the primary hypothesis that TEAS and acupressure can relieve CINV, and the secondary hypothesis that these two non-invasive TCM-based interventions can improve patients’ anxiety, depression and sleep quality, aiming to provide evidence for the clinical development of non-invasive strategies for comprehensive symptom management in such patients.

## 2 Methods/design

### 2.1 Study design

**Figure 1:**
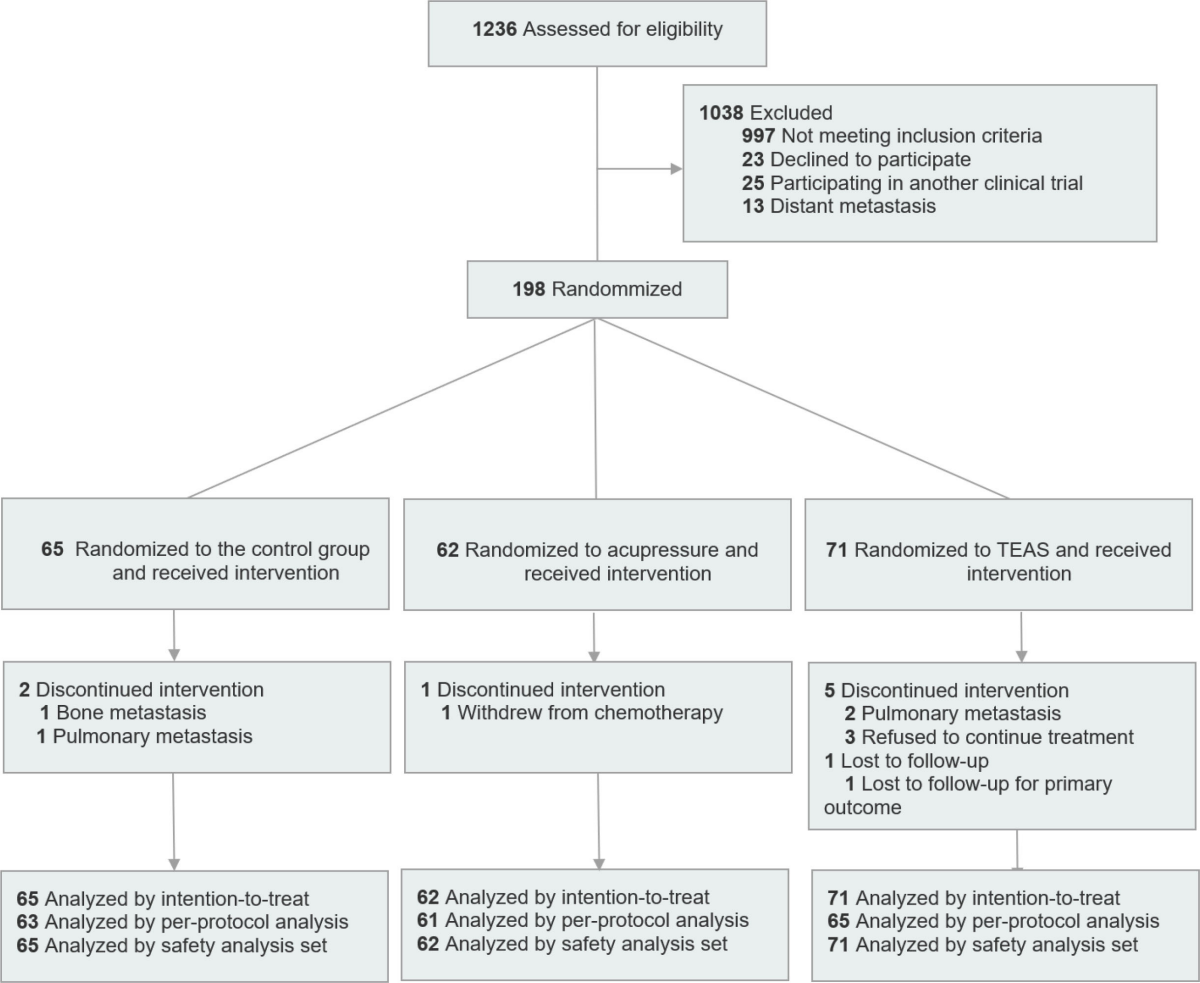
CONSORT flow diagram.

This was a single-center, three-arm parallel-group RCT with longitudinal follow-up. This study has completed pre-registration at the Chinese Clinical Trial Registry (Trial ID: ChiCTR2300077667 on 15 November 2023; available at https://www.chictr.org.cn/). The study was conducted in accordance with the Declaration of Helsinki and approved by the Ethics Committee of Affiliated Hospital of Chengdu University of Traditional Chinese Medicine (Approval No.: 2023KL-093). All participants provided written informed consent prior to enrollment. We followed the Consolidated Standards of Reporting Trials (CONSORT) reporting guideline for the design and reporting of this study (32) (see **Supplementary 1**). CONSORT flow diagram see **Figure 1**.

### 2.2 Participants

Participants were prospectively recruited from the Oncology Department of Sichuan Cancer Hospital between December 2023 and March 2025. Clinical data of breast cancer patients undergoing four cycles of chemotherapy were collected. Inclusion Criteria:(1) Female patients aged 18–70 years; (2) Patients with breast cancer meeting the diagnostic criteria;(3) Scheduled to receive chemotherapy; (4) No chemotherapy contraindications or liver/kidney function impairment. Exclusion Criteria:(1) Complicated with cardiac, liver, or kidney insufficiency, hematopoietic insufficiency, or other serious systemic diseases; (2) Nausea and vomiting caused by increased intracranial pressure, gastrointestinal diseases, or other non-chemotherapy-related factors; (3) Participation in other clinical trials involving behavioral, psychological, or complementary medical interventions during the study period; (4) History of trypanophobia (fear of acupuncture/puncture); (5) In pregnancy or lactation period; (6) Presence of mental or cognitive disorders that prevent normal communication. Withdrawal and Exclusion Criteria:(1) Poor compliance and refusal to cooperate with treatment; (2) Inability to continue treatment due to unexpected events during the intervention; (3) Voluntary withdrawal from the trial; (4) Lost to follow-up.

### 2.3 Randomization and blinding

Using a random number sequence generated by R 4.4.2, the sequence was stored in sealed, opaque envelopes numbered according to enrollment order by an independent researcher who was not involved in patient recruitment, treatment, or evaluation. When patients met the inclusion criteria and signed the informed consent form, the research coordinator opened the corresponding envelope in enrollment order to obtain the group assignment information.

Due to the specific nature of the interventions (TEAS requiring specialized equipment and acupressure being self-administered), blinding could not be implemented in this study. But the outcome assessors (including telephone follow-up staf) and data analysts were blinded to group allocation throughout the study, while only participants and intervention operators were unblinded due to the nature of non-pharmacological therapy.

### 2.4 Interventions

The specific locations of acupoints were determined in accordance with Nomenclature and Location of Acupoints issued by the National Technical Committee on Acupuncture and Moxibustion (33). The same set of acupoints was selected for both the acupressure group and the TEAS group, with bilateral intervention applied for both modalities: ①Hegu (LI4): On the dorsum of the hand, between the first and second metacarpal bones, approximately at the midpoint of the radial side of the second metacarpal bone; ②Neiguan (PC6): On the anterior aspect of the forearm, 2 cun above the distal transverse crease of the wrist, between the palmaris longus tendon and the flexor carpi radialis tendon; ③Zusanli (ST36): On the lateral aspect of the lower leg, 3 cun below Dubi (ST35), on the line connecting ST35 and Jiexi (ST41); ④Sanyinjiao (SP6): On the medial aspect of the lower leg, 6 cun above the tip of the medial malleolus, along the posterior border of the medial surface of the tibia.

#### 2.4.1 Control

Routine symptomatic treatment primarily antiemetic therapy was administered in accordance with the 2023 Update of the MASCC/ESMO Guidelines for the Prevention of Chemotherapy- and Radiotherapy-Induced Nausea and Vomiting issued by the Multinational Association of Supportive Care in Cancer (MASCC) and the European Society for Medical Oncology (ESMO) (34). For chemotherapy regimens with moderate-to-high emetogenic risk (including Cyclophosphamide + Epirubicin, Carboplatin + Docetaxel, and Carboplatin + Paclitaxel), a triple antiemetic prophylaxis regimen (NK-1 receptor antagonist + 5-HT3 receptor antagonist + dexamethasone) was administered. In contrast, for regimens with moderate-to-low emetogenic risk (including Doxorubicin Liposome+Cyclophosphamide and Docetaxel + Cyclophosphamide), dual antiemetic prophylaxis (5-HT3 receptor antagonist + dexamethasone) was used.

#### 2.4.2 Acupressure

In addition to routine symptomatic treatment, additional interventions were implemented as follows: ① On the day of baseline enrollment, the researchers demonstrated the acupressure techniques for the four selected acupoints to each participant. After confirming that the participants had mastered all key points such as acupoint localization, pressure intensity, and duration of pressing, the researchers added them on WeChat (the most popular chat APP in China) and sent acupressure demonstration videos. Participants were instructed to perform acupressure daily: once every morning and every evening, for 2–3 minutes per session, with the criterion of a soreness and distension sensation at the acupoints. Participants could consult the researchers via WeChat if they encountered any difficulties during acupressure. ②During the participants’ hospitalization, the researchers conducted daily face-to-face inquiries regarding their mastery of acupressure. Participants were re-advised to adhere to the daily acupressure regimen. ③On the 4th day after the completion of each chemotherapy cycle, telephone follow-ups were conducted to monitor the participants’ acupressure compliance. All interventions were performed by qualified trained physicians.

#### 2.4.3 TEAS

In addition to routine symptomatic treatment, the following additional intervention was implemented: Based on the patient’s hospital stay duration, TEAS was administered once daily on the day before chemotherapy, the day of chemotherapy, and the day after chemotherapy within each cycle, totaling 3 sessions per chemotherapy cycle. A Huatuo Brand transcutaneous electrical nerve stimulator (Model SDZ-II; Jiangsu Medical Device Registration Certificate No.: 20172200675) was used. The output current was set to 1–10 mA (adjusted according to the patient’s tolerance), the output waveform was a dense-disperse wave, the output frequency was 2/50 Hz, and each intervention session lasted 30 minutes. All interventions were performed by qualified trained physicians.

### 2.5 Measures

#### 2.5.1 Baseline characteristics

General information of participants, including age, ethnicity, educational level, marital status, occupation, medical insurance status, monthly income, and Body Mass Index (BMI), was collected through face-to-face interview-based questionnaires administered by researchers. History of disease-related risks (including smoking history, drinking history, and prior chemotherapy history) and disease-related information (including breast cancer subtype, whether surgical treatment was received, clinical stage, lymph node metastasis status, chemotherapy regimen, and antiemetic use) were retrieved from the Hospital Information System (HIS) after participants signed the informed consent form.

#### 2.5.2 Primary outcome

The incidence of CINV was evaluated using the MASCC Antiemesis Tool (MAT) on the 1st day and 5th day after chemotherapy completion in each cycle. Data on the 1st day were collected via face-to-face interview-based questionnaires, while data on the 5th day (after participants’ discharge) were obtained through telephone follow-ups. The MAT consists of 8 items (Cronbach’s α = 0.724) and assesses nausea and vomiting symptoms during both the acute phase (within 24 hours after chemotherapy) and the delayed phase (2–5 days after chemotherapy). These symptoms were converted into analyzable indicators (35, 36), including acute CINV, acute-phase nausea severity, delayed CINV, and delayed-phase nausea severity. Acute CINV was defined as a cumulative number of vomiting episodes ≥1 or a nausea level > 3 within 24 hours after chemotherapy; Delayed CINV was defined as a cumulative number of vomiting episodes ≥1 or a nausea level > 3 within 2–5 days after chemotherapy; The nausea severity was scored on a scale of 0 to 10 (0 = no nausea, 10 = most severe nausea).See **Supplementary 2**.

#### 2.5.3 Secondary outcomes

Data on secondary outcomes were collected on the day before the start of each chemotherapy cycle. See **Supplementary 2**.

##### 2.5.3.1 Sleep quality

Sleep quality was assessed using the Pittsburgh Sleep Quality Index (PSQI) (37). A total of 18 items were included in the scoring, which constitute 7 dimensions. Each dimension is scored on a range of 0–3 points, and the cumulative score of the 7 dimensions yields the total PSQI score (range: 0–21 points). A higher total score indicates poorer sleep quality (Cronbach’s α = 0.774).

##### 2.5.3.2 Anxiety and depression

Psychological distress was evaluated using the Hospital Anxiety and Depression Scale (HADS) (38). The scale comprises 2 subscales (Anxiety Subscale and Depression Subscale), each consisting of 7 items scored on a 4-point Likert scale (range: 0-3). The total score for each subscale ranges from 0 to 21 points, with higher scores indicating more severe anxiety or depression symptoms (Cronbach’s α = 0.826).

##### 2.5.3.3 Quality of life

QOL was assessed using the FACT-B questionnaire (39). Items are measured on a 5-point Likert scale ranging from 0 (“not at all”) to 4 (“very much so”). A higher total score indicates better QOL (Cronbach’s α = 0.873).

#### 2.5.4 Safety assessment

During the intervention period, local or systemic adverse reactions related to the interventions were recorded based on a combination of researcher observations and patient self-reports. These adverse reactions included local skin pain, tingling sensation, erythema and swelling, pruritus, skin allergy, local infection, and other systemic abnormal sensations.

### 2.6 Sample size calculation

The sample size was calculated using PASS 2023 software, with the delayed nausea severity score as the primary indicator. Preliminary pilot study results showed that the severity score of delayed nausea and vomiting was 3.58 in the control group and 2.38 in the TEAS group. The parameters were set as follows: standard deviation (σ) = 2, power (β) = 80%, significance level (α) = 0.05, and a two-tailed test. Based on these settings, 45 participants per group were required. Accounting for a potential 20% dropout rate, 57 participants should be enrolled per group, resulting in a total sample size of 171 participants. The formula is as follows:


$$n=\frac{2\sigma^{2}\left(Z_{1-\alpha /2}+Z_{1-\beta }\right)}{\delta }$$

### 2.7 Statistical analysis

A database was established using EpiData3.1 Software, and double data entry was implemented for all data to ensure accuracy. Data analysis was performed using SPSS Statistical Software (Version 26.0, IBM Corporation, USA) and R 4.4.2, with a two-tailed test and a significance level (α) set at 0.05. The Intention-to-Treat (ITT) principle was followed, and missing values were handled using Multiple Imputation by Chained Equations (MICE). For descriptive analysis: Continuous variables: Normally distributed data with homogeneous variances were presented as mean ± standard deviation ( $\bar{\text{x}}\pm \text{s}$); non-normally distributed data with heterogeneous variances were reported as median (M) and interquartile range (IQR); Categorical variables: Described using frequency (n) and constituent ratio (%). Between-group comparisons were conducted using Analysis of Variance (ANOVA)/Kruskal-Wallis test for continuous variables and Chi-square test/Fisher’s exact test for categorical variables.

The geepack package was loaded in R 4.4.2, and the geeglm function was invoked to perform Generalized Estimating Equations (GEE) analysis for evaluating intervention efficacy across different groups and chemotherapy cycles (40). The interaction effect between group and chemotherapy cycle was also considered, with covariates including baseline characteristics showing statistically significant between-group differences (*P*< 0.05). The link function was selected as “logit” (for dichotomous variables) or “gaussian” (for continuous variables), and the intraclass correlation structure was set to “ar1”. *Post-hoc* comparisons were conducted to identify differences in outcome indicators among groups within each chemotherapy cycle. Pairwise comparisons were performed using Empirical Marginal Means (EMMs) calculated from the parameter estimates of the GEE model. The emmeans function was called with adjust = “bonferroni” to adjust the significance level for controlling the risk of Type I error (41). Finally, the pairs () function was used to output the estimated means/rates, standard errors (SE), adjusted *P*-values, and 95% confidence intervals (95% CIs) for each comparison group.

Sensitivity analysis was performed using the per-protocol set (PPS) dataset.

## 3 Results

### 3.1 Patient characteristics

A total of 198 breast cancer patients who completed four cycles of chemotherapy were enrolled, including 65 in the control group, 62 in the acupressure group, and 71 in the TEAS group. During the study, there were 2 dropouts in the control group, 1 dropout in the acupressure group, and a total of 6 dropouts and exclusions in the TEAS group (see **Figure 1**).

In the ITT dataset, no statistically significant differences were observed in baseline characteristics among the groups, except for occupation category and type of antiemetic used, indicating good comparability (the results of baseline characteristic comparisons in the PPS dataset were consistent with those in the ITT dataset; see **Supplementary 3**). Detailed results are presented in **Table 1**.

**Table 1:** Baseline characteristics of the ITT population.

Variables	Total (n = 198)	Control (n = 65)	Acupressure (n = 62)	TEAS (n = 71)	χ^2^	*P*
Age, M (Q_1_, Q_3_)	50.00 (43.25, 55.00)	50.00 (42.00,56.00)	50.00 (45.00,55.00)	50.00 (43.00,55.00)	0.02#	0.991
n(%)						
Ethnicity					–	0.738
Han Chinese	190 (95.96)	63 (96.92)	60 (96.77)	67 (94.37)		
Ethnic minorities	8 (4.04)	2 (3.08)	2 (3.23)	4 (5.63)		
Marital Status					–	0.394
Married	184 (92.93)	59 (90.77)	60 (96.77)	65 (91.55)		
Divorced	10 (5.05)	5 (7.69)	2 (3.23)	3 (4.23)		
Unmarried	4 (2.02)	1 (1.54)	0 (0.00)	3 (4.23)		
Educational Level					10.68	0.220
Primary school or below	34 (17.17)	8 (12.31)	11 (17.74)	15 (21.13)		
Junior high school	81 (40.91)	26 (40.00)	28 (45.16)	27 (38.03)		
High school/vocational school	50 (25.25)	23 (35.38)	11 (17.74)	16 (22.54)		
College/university undergraduate	27 (13.64)	7 (10.77)	8 (12.90)	12 (16.90)		
Master’s degree or above	6 (3.03)	1 (1.54)	4 (6.45)	1 (1.41)		
Occupation					22.64	0.004
Worker/Farmer	37 (18.69)	14 (21.54)	7 (11.29)	16 (22.54)		
Commercial/service industry practitioner	39 (19.70)	7 (10.77)	20 (32.26)	12 (16.90)		
Staff of public institutions/Civil servant	57 (28.79)	18 (27.69)	21 (33.87)	18 (25.35)		
Freelancer	29 (14.65)	7 (10.77)	6 (9.68)	16 (22.54)		
Others (e.g., retired, unemployed)	36 (18.18)	19 (29.23)	8 (12.90)	9 (12.68)		
Monthly Income					12.92	0.228
<1000 RMB	17 (8.59)	3 (4.62)	5 (8.06)	9 (12.68)		
1000–1999 RMB	66 (33.33)	25 (38.46)	20 (32.26)	21 (29.58)		
2000–2999 RMB	32 (16.16)	5 (7.69)	11 (17.74)	16 (22.54)		
3000–3999 RMB	49 (24.75)	18 (27.69)	16 (25.81)	15 (21.13)		
4000–4999 RMB	17 (8.59)	5 (7.69)	7 (11.29)	5 (7.04)		
≥5000 RMB	17 (8.59)	9 (13.85)	3 (4.84)	5 (7.04)		
Medical Insurance Status					–	0.185
Self-payment	2 (1.01)	1 (1.54)	0 (0.00)	1 (1.41)		
Urban-rural resident basic medical insurance	112 (56.57)	31 (47.69)	35 (56.45)	46 (64.79)		
Urban employee basic medical insurance	84 (42.42)	33 (50.77)	27 (43.55)	24 (33.80)		
Smoking History					–	0.624
No	193 (97.47)	64 (98.46)	61 (98.39)	68 (95.77)		
Yes	5 (2.53)	1 (1.54)	1 (1.61)	3 (4.23)		
Drinking History					–	1.000
No	194 (97.98)	64 (98.46)	61 (98.39)	69 (97.18)		
Yes	4 (2.02)	1 (1.54)	1 (1.61)	2 (2.82)		
Cancer Subtype					–	0.683
Papillary tumor	1 (0.51)	0 (0.00)	0 (0.00)	1 (1.41)		
Ductal carcinoma *in situ* (DCIS)	3 (1.52)	1 (1.54)	1 (1.61)	1 (1.41)		
Invasive carcinoma	172 (86.87)	59 (90.77)	55 (88.71)	58 (81.69)		
Invasive carcinoma with ductal carcinoma in situ	22 (11.11)	5 (7.69)	6 (9.68)	11 (15.49)		
Prior Chemotherapy History					–	0.649
No	195 (98.48)	65 (100.00)	61 (98.39)	69 (97.18)		
Yes	3 (1.52)	0 (0.00)	1 (1.61)	2 (2.82)		
Clinical Stage					1.32	0.857
Stage I	53 (26.77)	15 (23.08)	17 (27.42)	21 (29.58)		
Stage II	114 (57.58)	41 (63.08)	35 (56.45)	38 (53.52)		
Stage III	31 (15.66)	9 (13.85)	10 (16.13)	12 (16.90)		
Lymph Node Metastasis					0.98	0.614
No	121 (61.11)	40 (61.54)	35 (56.45)	46 (64.79)		
Yes	77 (38.89)	25 (38.46)	27 (43.55)	25 (35.21)		
Chemotherapy Regimen					6.98	0.137
Low emetic risk regimen	39 (19.70)	14 (21.54)	15 (24.19)	10 (14.08)		
Moderate emetic risk regimen	67 (33.84)	19 (29.23)	26 (41.94)	22 (30.99)		
High emetic risk regimen	92 (46.46)	32 (49.23)	21 (33.87)	39 (54.93)		
Antiemetic Agent					28.27	<0.001
5-HT_3_ receptor antagonist	67 (33.84)	13 (20.00)	13 (20.97)	41 (57.75)		
5-HT_3_ + NK-1 receptor antagonist	131 (66.16)	52 (80.00)	49 (79.03)	30 (42.25)		
BMI Category					–	0.677
BMI<18.5	7 (3.54)	3 (4.62)	2 (3.23)	2 (2.82)		
18.5≤BMI<24.0	100 (50.51)	28 (43.08)	35 (56.45)	37 (52.11)		
24.0≤BMI<28.0	66 (33.33)	25 (38.46)	16 (25.81)	25 (35.21)		
BMI≥28.0	25 (12.63)	9 (13.85)	9 (14.52)	7 (9.86)		
Surgical Status					0.87	0.646
No	98 (49.49)	34 (52.31)	32 (51.61)	32 (45.07)		
Yes	100 (50.51)	31 (47.69)	30 (48.39)	39 (54.93)		
Number of Chemotherapy Cycles					0.39	0.822
Four cycles	47 (23.74)	16 (24.62)	13 (20.97)	18 (25.35)		
Six cycles	151 (76.26)	49 (75.38)	49 (79.03)	53 (74.65)		
M (Q_1_, Q_3_)						
Baseline PSQI	6.00 (4.00, 9.00)	6.00 (4.00,9.00)	6.00 (4.00,9.00)	5.00 (4.00,8.00)	0.22#	0.896
Baseline HADS-D	5.00 (2.00, 7.00)	5.00 (2.00,7.00)	5.00 (2.00,6.75)	4.00 (2.00,6.00)	2.44#	0.296
Baseline HADS-A	4.50 (2.00, 7.00)	5.00 (3.00,8.00)	4.00 (2.00,6.75)	4.00 (2.00,6.00)	4.00#	0.135
Baseline FACT-B	107.00(95.00, 116.00)	101.00(92.00, 114.50)	105.00(97.00, 118.00)	109.00(97.00, 116.00)	2.15#	0.431

#, Kruskal-Wallis test; -, Fisher exact; M, Median; Q_1_, 1st Quartile; Q_3_, 3rd Quartile.

### 3.2 Analysis of therapeutic effect and sensitivity

Primary Outcome: In the ITT analysis, the risk of delayed CINV in Cycle 3 was higher than that in Cycle 1. Compared with the control group, the TEAS group showed a decreasing trend in the risk of delayed CINV and delayed nausea severity in Cycle 4. No statistically significant differences in acute nausea severity were observed among the TEAS group, acupressure group, and control group (all *P*>0.05). In the PPS analysis, the TEAS group showed a decreasing trend in the risk of acute CINV in Cycle 3. All other results regarding CINV were consistent with the ITT analysis in direction (see **Table 2**).

**Table 2:** Results of ITT data analysis using GEE.

Outcome	Variables	ITT	PPS
		OR (95% CI)/ β (95% CI)	OR (95% CI)/ β (95% CI)
Acute CINV			
	TEAS *cycle3	-	0.371 (0.146-0.943)
Acutenausea	-	-	-
Delayed CINV			
	cycle3vs.cycle1	1.870 (1.067, 3.278)	1.785 (1.013, 3.147)
	TEAS *cycle4	0.424 (0.183, 0.985)	0.369 (0.156, 0.874)
Delayed nausea			
	TEAS *cycle4	-1.256 (-2.465, -0.047)	-1.492 (-2.707, -0.277)
Sleep Quality			
	TEAS *cycle3	-1.581 (-3.141, -0.021)	-1.592 (-3.144, -0.040)
	TEAS *cycle4	–	-1.636 (-3.239, -0.033)
Anxiety			
	Acupressure vs. Control	-1.382 (-2.473, -0.291)	-1.246 (-2.330, -0.162)
	cycle3vs.cycle1	-0.969 (-1.796, -0.142)	-0.984 (-1.817, -0.151)
	5-HT_3_+NK-1 receptor antagonist vs. 5-HT_3_ receptor antagonist	0.729 (0.045, 1.413)	–
	TEAS *cycle4	-1.217 (-2.416, -0.018)	-1.636 (-3.239, -0.033)
Depression			
	Acupressure vs. Control	-0.980 (-1.932, -0.028)	–
	staff of public institutions/civil servants vs. Worker/Farmer	–	0.898 (0.005, 1.791)
	TEAS *cycle4	-1.305 (-2.405, -0.205)	-1.505 (-2.591, -0.419)
Quality of Life			
	TEAS vs. Control	5.286 (1.062, 9.510)	5.333 (0.957, 9.709)
	TEAS *cycle2	4.541 (0.666, 8.416)	4.826 (0.886, 8.766)
	TEAS *cycle3	4.198 (0.160, 8.236)	4.820 (0.847, 8.793)
	Acupressure *cycle4	6.809 (2.131, 11.487)	6.824 (2.146, 11.502)
	TEAS *cycle4	7.852 (3.699, 12.005)	8.513 (4.366, 12.660)

“-” indicates that there was no statistically significant difference in the covariates included in the model for this outcome;

Outcome indicators: Acute CINV, severity of nausea during the acute phase, delayed CINV, severity of nausea during the delayed phase, sleep quality, anxiety, depression, quality of life;

Fixed effect variables: Group, chemotherapy cycle;

Covariates: Occupation, type of antiemetic medication taken, interaction effect between group and chemotherapy cycle.

The GEE model used an AR1 (autoregressive order 1) autocorrelation structure, considering the correlation of adverse reactions between consecutive chemotherapy cycles. Gaussian distribution link function was applied for continuous variables, and logit link function for binary variables. Covariates were selected due to their baseline imbalance to reduce potential confounding. Detailed original GEE model results are presented in **Supplementarys 4** and **5**.

Secondary Outcomes: For sleep quality, the TEAS group exhibited an improving trend in Cycle 3 in the ITT analysis, and a decreasing trend in sleep quality scores in Cycle 4 in the PPS analysis. Regarding psychological state, anxiety levels in Cycle 3 were lower than those in Cycle 1. The acupressure group showed a significant main effect of group on anxiety and depression. Compared with the control group, the TEAS group presented an improving trend in anxiety and depression levels in Cycle 4. The use of 5−HT_3_ + NK−1 receptor antagonists was associated with increased anxiety levels, while occupation as staff of public institutions or civil servants was related to elevated depression levels. For QOL, the TEAS group showed a significant main effect of group, with scores displaying an increasing trend in Cycles 2, 3, and 4. The acupressure group also exhibited an increasing trend in QOL scores in Cycle 4.

Intervention−by−Time Interaction Effects: Significant intervention−by−time interaction effects were observed for delayed CINV, delayed nausea severity, sleep quality, anxiety, depression, and QOL, with notable changes observed mainly across Cycles 3 and 4.

Overall, the results of the PPS analysis were consistent with those of the ITT analysis in directional trends (see **Table 2**). Detailed results of the original data analysis are presented in **Supplementary 4** and **Supplementary 5**.

### 3.3 Post-event comparison and sensitivity analysis

In the ITT analysis, compared with the control group, the TEAS group was more effective in controlling the risk of delayed CINV, improving sleep quality in Cycle 3 and Cycle 4, alleviating delayed nausea, reducing anxiety and depression levels in Cycle 4, and enhancing QOL across all cycles. When compared with the acupressure group, the TEAS group showed superior performance in controlling delayed CINV risk in Cycle 3 and improving QOL in Cycle 4. In contrast, the acupressure group outperformed the control group in reducing anxiety levels in Cycle 1, alleviating delayed nausea, decreasing anxiety and depression levels, and improving QOL in Cycle 4; additionally, the acupressure group exhibited a more significant effect in relieving anxiety and depression in Cycle 1 compared with the TEAS group. No statistically significant differences in acute nausea severity were observed among the three groups (all *P* > 0.05).

In the PPS analysis, occupation as staff of public institutions/civil servants was associated with increased depression levels; the TEAS group showed a decreasing trend in acute CINV risk in Cycle 3 and a decreasing trend in sleep quality scores in Cycle 4, and was superior to the acupressure group in QOL in Cycle 2 and Cycle 3 as well as sleep quality in Cycle 4. Furthermore, the acupressure group had a lower risk of acute CINV in Cycle 4 compared with the control group, and all other results were consistent with the ITT analysis in terms of direction. See **Table 3** for comprehensive results.

**Table 3:** Results of *Post-hoc* comparison analysis of GEE model.

Number of chemotherapy cycles	Outcome	Comparison	ITT	PPS
			OR(95%CI) /β (95% CI)	OR(95%CI) /β (95% CI)
1				
	Anxiety	Acupressure vs Control	-1.382 (-2.716, -0.048)	–
	Anxiety	TEAS vs Acupressure	1.682 (0.170, 3.194)	1.590 (0.025, 3.155)
	Depression	TEAS vs Acupressure	1.391 (0.119, 2.663)	1.437 (0.134, 2.740)
	Quality of Life	TEAS vs Control	5.290 (0.116, 10.464)	5.333 (0.957, 9.709)
2				
	Quality of Life	TEAS vs Control	9.830 (4.438, 15.210)	10.159 (4.668, 15.650)
	Quality of Life	TEAS vs Acupressure	–	5.525 (0.279, 10.771)
3				
	Delayed CINV	TEAS vs Control	0.296 (0.115, 0.764)	0.296 (0.115, 0.761)
	Delayed CINV	TEAS vs Acupressure	0.359 (0.138, 0.929)	0.331 (0.135, 0.814)
	Sleep Quality	TEAS vs Control	-2.008 (-3.800, -0.217)	-1.995 (-3.761, -0.229)
	Quality of Life	TEAS vs Control	9.480 (4.661, 14.310)	10.150 (5.461, 14.839)
	Quality of Life	TEAS vs Acupressure	–	5.904 (1.033, 10.775)
4				
	Acute CINV	Acupressure vs Control	–	0.282 (0.088, 0.913)
	Delayed CINV	TEAS vs Control	0.245 (0.095, 0.635)	0.213 (0.080, 0.566)
	Delayed nausea	Acupressure vs Control	-1.425 (-2.725, -0.125)	-1.368 (-2.698, -0.039)
	Delayed nausea	TEAS vs Control	-1.547 (-2.904, -0.189)	-1.730 (-3.106, -0.354)
	Sleep Quality	TEAS vs Control	-2.078 (-3.740, -0.417)	-2.039 (-3.635, -0.443)
	Sleep Quality	TEAS vs Acupressure	–	-1.563 (-3.050, -0.076)
	Anxiety	Acupressure vs Control	-1.730 (0.591, 2.869)	-1.626 (-2.750, -0.502)
	Depression	Acupressure vs Control	-1.253 (-2.223, -0.282)	-1.110 (-2.066, -0.154)
	Quality of Life	Acupressure vs Control	8.060 (3.404, 12.710)	7.720 (3.081, 12.359)
	Quality of Life	TEAS vs Control	13.140 (8.105, 18.170)	13.846 (8.888, 18.804)
	Quality of Life	TEAS vs Acupressure	5.080 (0.778, 9.380)	6.126 (1.890, 10.362)

“-” indicates that there was no statistically significant difference for this outcome. All pairwise comparisons were adjusted using the Bonferroni method.

**Figure 2:**
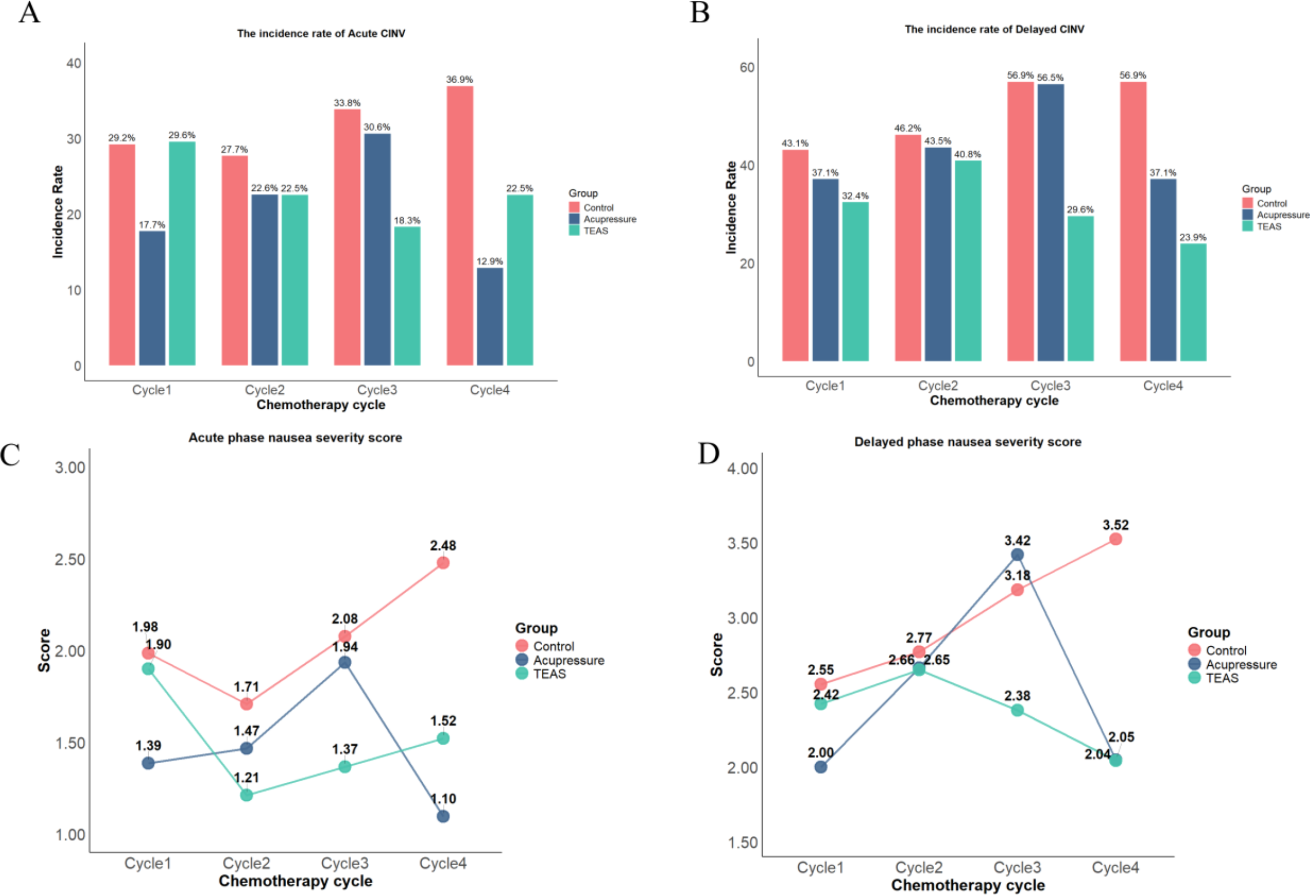
Occurrence of each chemotherapy cycle for the primary outcome. **(A)** Incidence of Acute CINV; **(B)** Incidence of Delayed CINV; **(C)** Acute Nausea; **(D)** Delayed Nausea Score.

**Figure 3:**
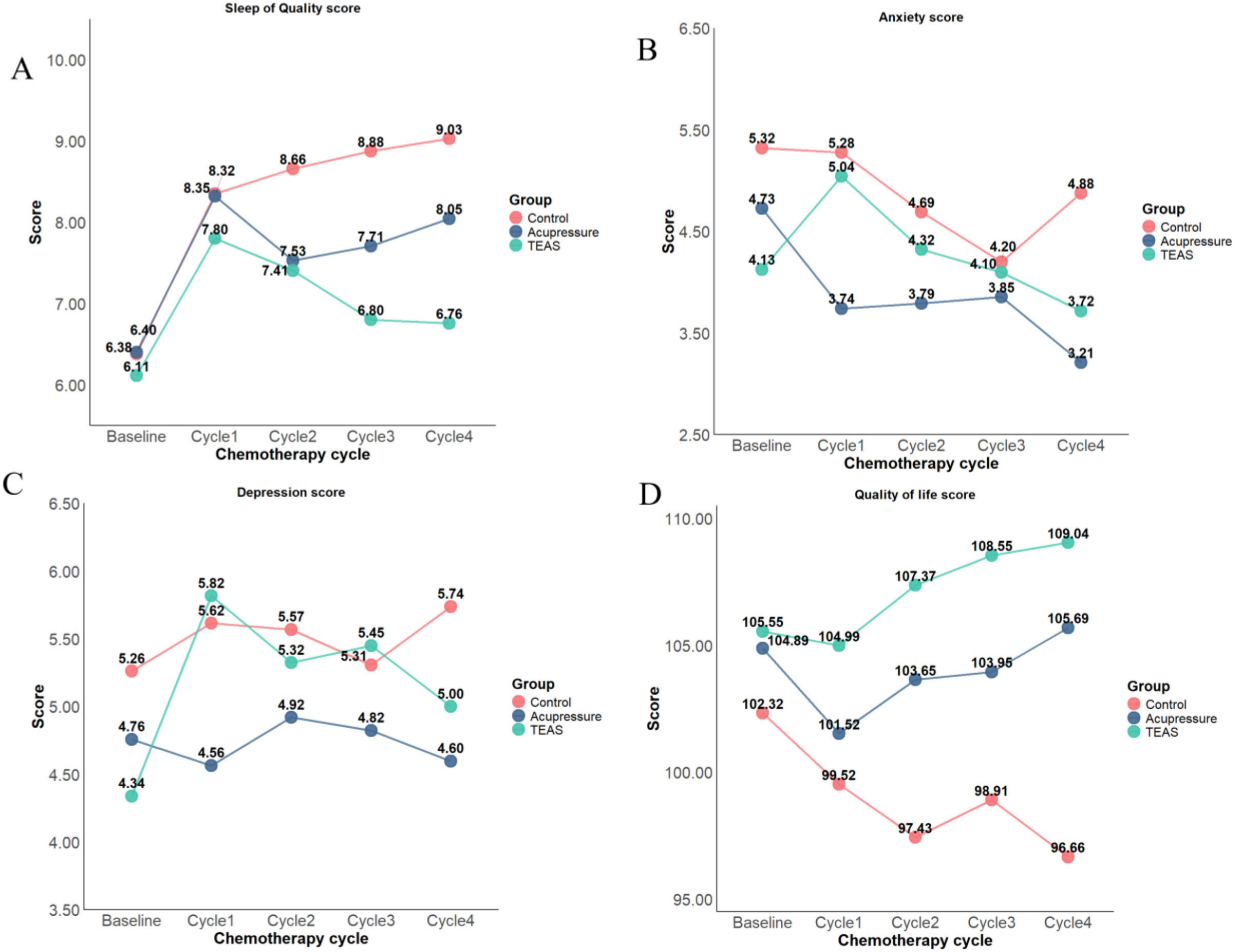
Occurrence of secondary outcome during each chemotherapy cycle. **(A)** Sleep Quality; **(B)** Anxiety; **(C)** Depression; **(D)** Quality of Life. Higher scores indicate better QOL, but worse sleep quality and more severe anxiety/depression symptom).

Detailed values of each indicator across all chemotherapy cycles are presented in **Figure 2** and **Figure 3**.

## 4 Safety assessment

Overall, no local adverse reactions such as skin pain, tingling, erythema, swelling, pruritus, skin allergy, or local infection were observed in any participant. Regarding systemic abnormal sensations, two cases were reported in the TEAS group: one patient experienced palpitations during the second cycle of chemotherapy, and another patient experienced palpitations during the third cycle. In both cases, the palpitations occurred within one minute of initiating the TEAS intervention and were quickly relieved by the attending staff. No other systemic abnormal sensations or adverse reactions were reported in the TEAS, acupressure, or control groups throughout the study period.

## 5 Discussion

The results of this study indicated that TEAS and acupressure exerted dynamic and targeted improvement effects on multiple symptoms in breast cancer patients undergoing chemotherapy. The main effect of time showed that with the progression of chemotherapy, the risk of delayed CINV increased significantly, suggesting that the cumulative effect of chemotherapy toxicity was more pronounced in the later stages. Patients’ anxiety levels decreased after Cycle 3, which was consistent with previous studies (42) —anxiety levels are highest at the time of diagnosis and the initial stage of treatment, and tend to decrease during the treatment process. The main effect of TEAS intervention was reflected in improving QOL, while the main effect of acupressure was manifested in alleviating psychological symptoms such as anxiety and depression. The higher anxiety levels observed in patients who received 5−HT_3_ + NK−1 receptor antagonist antiemetic treatment may reflect an observational correlation rather than a causal effect of the medication. Neurobiologically, the expression of NK−1 receptors in the amygdala has been reported to be associated with individual anxiety−related traits, which may partly explain this correlational finding (9). In this study, all patients who received moderate-to-highly emetogenic chemotherapy regimens, such as Cyclophosphamide + Epirubicin, Carboplatin + Docetaxel, and Carboplatin + Paclitaxel, were administered NK-1 receptor antagonist therapy for the prevention of nausea and vomiting. Notably, such highly emetogenic regimens induce patients ‘high anxiety susceptibility and disease-related stress, which not only necessitated intensive antiemetic regimens but also led to increased anxiety levels (43). In the PPS analysis, patients with occupations as staff of public institutions/civil servants had elevated depression levels. This may be attributed to the fact that this group has higher health expectations and needs; the diagnosis of breast cancer disrupts the stability and predictability of their career development paths, resulting in a sense of loss of control, uncertainty, and disparity, which are more likely to induce depressive emotions. However, no statistically significant difference was observed in the ITT analysis (*P* > 0.05), and further research is needed to verify the stability of this conclusion.

Analysis of the interaction effect between intervention and chemotherapy cycle revealed that the improving effects of TEAS on the risk of delayed CINV, sleep quality, and delayed nausea in the middle and late stages of chemotherapy (Cycles 3–4) may be associated with its cumulative therapeutic effect. Multiple electrical stimulations can gradually regulate the meridian qi of the spleen and stomach; particularly in the later stages of chemotherapy, when patients have a debilitated physical condition and insufficient healthy qi, the improvement effect becomes more prominent. From a modern medical perspective, such cumulative effects may be attributed to the repeated regulation of neurotransmitters (e.g., β-endorphin and serotonin) and the activation of anti-inflammatory pathways following repetitive electrical stimulation, which may lead to gradually enhanced therapeutic effects over multiple intervention cycles (20, 44). This further strengthens the synergistic effect of “descending counterflow to stop vomiting” among various acupoints, alleviates patients’ anxiety and depression symptoms, and improves their QOL. Although there is no consensus on the specific minimum clinically important difference(MCID) for the FACT-B scale among breast cancer patients undergoing chemotherapy, published studies have confirmed that the MCID for the FACT series of scales in the cancer patient population is typically between 4 and 10 points (45, 46). In this study, the TEAS group and the acupressure group each increased the total FACT-B score by 3 to 4 points during the chemotherapy cycle, while the control group decreased by 5.56 points. Although the improvement amplitudes of the two intervention groups did not fully exceed the upper limit of the traditional MCID range, the differences between the groups (improvement in the intervention group and deterioration in the control group) were significant and clinically meaningful.

The therapeutic effect of acupressure varies across different stages: it exhibits a good effect in relieving anxiety in the early stage of chemotherapy (Cycle 1), and can simultaneously alleviate delayed nausea and improve QOL in the later stage. As a low-threshold self-management strategy, acupressure can effectively provide psychological support in the initial stage of treatment. With the improvement of patients’ proficiency, the benefits for somatic responses continue to accumulate, and the QOL increases as patients accept their condition, making the overall benefits of acupressure more prominent in the later stage.

According to TCM, the occurrence of a series of symptoms such as nausea and vomiting, anxiety and depression, sleep disorders, and decreased QOL in breast cancer patients undergoing chemotherapy is primarily attributed to the core pathogenesis of “deficiency in root and excess in branch” with multiple disorders (13, 47). “Deficiency in root” refers to the severe consumption of qi and blood by chemotherapeutic toxins, leading to qi deficiency of the spleen and stomach, yin deficiency of the liver and kidney, and insufficient nourishment of the heart. “Excess in branch” is characterized by internal accumulation of chemotherapeutic toxins, upward adverse flow of stomach qi, and stagnation of liver qi. The therapeutic principle should focus on “invigorating the spleen and harmonizing the stomach, descending counterflow to stop vomiting”, while also considering “soothing the liver to relieve stagnation, regulating qi and blood, and nourishing the heart to tranquilize the mind”. Within this pathogenic framework, the acupoint selection in this study embodies the TCM principle of “treating both the root and the branch” (31, 48, 49). Zusanli (ST36) and Hegu (LI4) are respectively the “He-sea point” of the Stomach Meridian of Foot-Yangming and the “Yuan-source point” of the Large Intestine Meridian of Hand-Yangming. The Yangming meridians are abundant in qi and blood, and their pathways pass through the stomach and belong to the intestines. The combination of these two points regulates the intestines and stomach, strengthens the spleen and stomach, and serves as a key method for restoring the transportation and transformation function of the middle jiao (triple energizer) to promote the production of qi and blood, representing an essential “supporting healthy qi” strategy. Neiguan (PC6) is the “Luo-connecting point” of the Pericardium Meridian of Hand-Jueyin and also connects to the Yinwei Meridian. The Pericardium Meridian “circulates and connects the triple jiao”, while the Yinwei Meridian “governs the interior of the whole body”; thus, Neiguan possesses the effects of relieving chest stuffiness and regulating qi, harmonizing the stomach and descending counterflow, and tranquilizing the mind. Sanyinjiao (SP6) is an important point of the Spleen Meridian of Foot-Taiyin and the confluent point of the three yin meridians of the foot (liver, spleen, and kidney), regulating three meridians through a single point. Its functions include invigorating the spleen and benefiting the stomach to tonify the acquired constitution, as well as nourishing water to moisten wood and regulating the liver and kidney. Through the methods of distant-near point combination and simultaneous treatment of exterior-interior meridians, these acupoints collectively exert comprehensive effects of harmonizing the stomach to descend counterflow, invigorating the spleen to replenish qi, soothing the liver to relieve stagnation, and nourishing blood to tranquilize the mind.

This study showed that the therapeutic effect of TEAS enhanced with the progression of chemotherapy cycles, particularly showing significant improvements in acute CINV, delayed CINV, sleep quality, and anxiety and depression in the later stages. This corresponds to the TCM theory of “prolonged illness affecting the kidney” and “gradual recovery of healthy qi”. With the accumulation of interventions, the qi of the spleen and stomach is activated, ensuring a source of qi and blood production; liver stagnation is relieved, and the mind is nourished. Thus, the improvement effect can still be manifested in the later stages of cumulative chemotherapy toxicity. Compared with the control group, acupressure demonstrated advantages in relieving anxiety, reflecting that its effect of “soothing the liver to relieve stagnation” can regulate qi movement and calm emotions in the early stage of intervention. The temporal difference in the emergence of therapeutic effects precisely reflects the different focuses and synergistic effects of the two intervention methods in treating the “branch” and “root”.

Similar to previous studies (12, 22, 24), this study confirmed that TEAS and acupressure can effectively alleviate adverse reactions such as nausea, anxiety, depression, and decreased sleep quality during chemotherapy, and improve patients’ QOL. The difference lies in that most previous studies adopted a single-cycle design or self-defined observation time points (15, 22, 23, 50), leading to inconsistent conclusions on the intervention efficacy of TEAS and acupressure. In contrast, this study implemented full-cycle observation, which started from the first chemotherapy cycle and continued until the end of the entire chemotherapy course. Interventions and follow-ups were conducted simultaneously, which is consistent with real-world clinical chemotherapy scenarios. This design enabled dynamic evaluation of the improvement of patients’ adverse reactions across different chemotherapy cycles, thereby enhancing the authenticity and reliability of the conclusions.

## 6 Limitations

This study adopted a single-center design. Future studies should consider conducting multi-center clinical trials to improve the external validity of the conclusions. Additionally, since the intervention was implemented in a real-world clinical setting, blinding could not be implemented, and further research is needed to verify the placebo effect associated with the intervention outcomes. Notably, the lack of blinding, a common challenge in non-pharmacological trials, may be a potential source of bias. Finally, Although Bonferroni correction was applied in *post-hoc* analyses, the potential inflation of type I error due to multiple outcomes, time points, and comparisons cannot be completely excluded, and thus the secondary outcomes should be interpreted with caution.

## 7 Conclusion

TEAS and acupressure are effective complementary therapies for alleviating chemotherapy-related symptoms in breast cancer patients. TEAS exerts a significant effect in the middle and late stages of chemotherapy, reducing the risk of delayed CINV, improving anxiety, depression, sleep quality, and QOL through a cumulative effect. In contrast, acupressure effectively alleviates anxiety in the early stage of chemotherapy and improves nausea symptoms and QOL in the later stage. Compared with previous studies, the longitudinal design of this study is more consistent with clinical practice, providing evidence-based support for the application of these two therapies in real-world settings.

## Data Availability

The raw data supporting the conclusions of this article will be made available by the authors, without undue reservation.
