# Supplementary material for: Efficacy and safety of transcutaneous electrical acupoint stimulation and acupressure in alleviating chemotherapy-related adverse reactions in female patients with breast cancer: a randomized clinical trial
Source: Front Oncol. 2026 Apr 15;16:1788635. doi: 10.3389/fonc.2026.1788635 (PMC13124625; doi:10.3389/fonc.2026.1788635)
Supplement: Supplementary file 2 [file Table2.docx]

| Variable Type | Measurement Tool (Reliability) | Time Point of Measurement | Content / Definition | Scoring / Interpretation |
| --- | --- | --- | --- | --- |
| **Primary Outcome** | **MASCC Antiemesis Tool (MAT)** (Cronbach's α = 0.724) | **Day 1**: Within 24 hours post‑chemotherapy (face‑to‑face interview). **Day 5**: 2–5 days post‑chemotherapy (delayed phase; telephone follow‑up). | • **Acute CINV**: ≥1 vomiting episode **or** nausea level > 3 within 24 h after chemotherapy. • **Acute nausea severity**: 0–10 scale (0 = no nausea, 10 = worst nausea). • **Delayed CINV**: ≥1 vomiting episode **or** nausea level > 3 within 2–5 d after chemotherapy. • **Delayed nausea severity**: 0–10 scale. | • Incidence of acute/delayed CINV is reported as proportion of patients meeting criteria. • Nausea severity: higher scores indicate more severe nausea. |
| **Secondary Outcomes** | **Pittsburgh Sleep Quality Index (PSQI)** (Cronbach's α = 0.774) | Day before each chemotherapy cycle. | 18 items covering 7 components of sleep quality (e.g., subjective quality, latency, duration). | Each component scored 0–3; global score range 0–21. **Higher scores = poorer sleep quality**. |
|  | **Hospital Anxiety and Depression Scale (HADS)** (Cronbach's α = 0.826) | Day before each chemotherapy cycle. | Two subscales: Anxiety (7 items) and Depression (7 items). 4‑point Likert scale (0–3). | Each subscale total 0–21. **Higher scores = more severe anxiety/depression**. |
|  | **Functional Assessment of Cancer Therapy – Breast (FACT-B)** (Cronbach's α = 0.873) | Day before each chemotherapy cycle. | Multi‑dimensional quality of life questionnaire for breast cancer patients. | 5‑point Likert scale (0 = not at all, 4 = very much). **Higher total score = better QOL**. |
