# Supplementary material for: Efficacy and safety of transcutaneous electrical acupoint stimulation and acupressure in alleviating chemotherapy-related adverse reactions in female patients with breast cancer: a randomized clinical trial
Source: Front Oncol. 2026 Apr 15;16:1788635. doi: 10.3389/fonc.2026.1788635 (PMC13124625; doi:10.3389/fonc.2026.1788635)
Supplement: Supplementary file 3 [file Table3.docx]

| Variables | Total (n = 189) | Control (n = 63) | Acupressure  (n = 61) | TEAS(n = 65) | χ² | *P* |
| --- | --- | --- | --- | --- | --- | --- |
| Age, M (Q₁, Q₃) | 50.00 (44.00, 55.00) | 50.00 (42.00,56.00) | 50.00 (45.00,55.00) | 50.00 (46.00,55.00) | 0.06# | 0.969 |
| n(%) | | | | | | |
| Ethnicity |  |  |  |  | - | 1.000 |
| Han Chinese | 182 (96.30) | 61 (96.83) | 59 (96.72) | 62 (95.38) |  |  |
| Ethnic minorities | 7 (3.70) | 2 (3.17) | 2 (3.28) | 3 (4.62) |  |  |
| Marital Status |  |  |  |  | - | 0.552 |
| Married | 176 (93.12) | 57 (90.48) | 59 (96.72) | 60 (92.31) |  |  |
| Divorced | 10 (5.29) | 5 (7.94) | 2 (3.28) | 3 (4.62) |  |  |
| Unmarried | 3 (1.59) | 1 (1.59) | 0 (0.00) | 2 (3.08) |  |  |
| Educational Level |  |  |  |  | 11.37 | 0.181 |
| Primary school or below | 31 (16.40) | 7 (11.11) | 11 (18.03) | 13 (20.00) |  |  |
| Junior high school | 78 (41.27) | 25 (39.68) | 28 (45.90) | 25 (38.46) |  |  |
| High school/vocational school | 48 (25.40) | 23 (36.51) | 10 (16.39) | 15 (23.08) |  |  |
| College/university undergraduate | 26 (13.76) | 7 (11.11) | 8 (13.11) | 11 (16.92) |  |  |
| Master's degree or above | 6 (3.17) | 1 (1.59) | 4 (6.56) | 1 (1.54) |  |  |
| Occupation |  |  |  |  | 22.88 | **0.004** |
| Worker/Farmer | 33 (17.46) | 13 (20.63) | 7 (11.48) | 13 (20.00) |  |  |
| Commercial/service industry practitioner | 37 (19.58) | 6 (9.52) | 19 (31.15) | 12 (18.46) |  |  |
| Staff of public institutions/Civil servant | 55 (29.10) | 18 (28.57) | 21 (34.43) | 16 (24.62) |  |  |
| Freelancer | 29 (15.34) | 7 (11.11) | 6 (9.84) | 16 (24.62) |  |  |
| Others (e.g., retired, unemployed) | 35 (18.52) | 19 (30.16) | 8 (13.11) | 8 (12.31) |  |  |
| Monthly Income |  |  |  |  | 14.43 | 0.154 |
| <1000 RMB | 17 (8.99) | 3 (4.76) | 5 (8.20) | 9 (13.85) |  |  |
| 1000-1999 RMB | 61 (32.28) | 24 (38.10) | 20 (32.79) | 17 (26.15) |  |  |
| 2000-2999 RMB | 32 (16.93) | 5 (7.94) | 11 (18.03) | 16 (24.62) |  |  |
| 3000-3999 RMB | 46 (24.34) | 17 (26.98) | 15 (24.59) | 14 (21.54) |  |  |
| 4000-4999 RMB | 16 (8.47) | 5 (7.94) | 7 (11.48) | 4 (6.15) |  |  |
| ≥5000 RMB | 17 (8.99) | 9 (14.29) | 3 (4.92) | 5 (7.69) |  |  |
| Medical Insurance Status |  |  |  |  | - | 0.210 |
| Self-payment | 2 (1.06) | 1 (1.59) | 0 (0.00) | 1 (1.54) |  |  |
| Urban-rural resident basic medical insurance | 107 (56.61) | 30 (47.62) | 35 (57.38) | 42 (64.62) |  |  |
| Urban employee basic medical insurance | 80 (42.33) | 32 (50.79) | 26 (42.62) | 22 (33.85) |  |  |
| Smoking History |  |  |  |  | - | 0.622 |
| No | 184 (97.35) | 62 (98.41) | 60 (98.36) | 62 (95.38) |  |  |
| Yes | 5 (2.65) | 1 (1.59) | 1 (1.64) | 3 (4.62) |  |  |
| Drinking History |  |  |  |  | - | 1.000 |
| No | 185 (97.88) | 62 (98.41) | 60 (98.36) | 63 (96.92) |  |  |
| Yes | 4 (2.12) | 1 (1.59) | 1 (1.64) | 2 (3.08) |  |  |
| Cancer Subtype |  |  |  |  | - | 0.529 |
| Papillary tumor | 1 (0.53) | 0 (0.00) | 0 (0.00) | 1 (1.54) |  |  |
| Ductal carcinoma in situ (DCIS) | 3 (1.59) | 1 (1.59) | 1 (1.64) | 1 (1.54) |  |  |
| Invasive carcinoma | 163 (86.24) | 57 (90.48) | 54 (88.52) | 52 (80.00) |  |  |
| Invasive carcinoma with ductal carcinoma in situ | 22 (11.64) | 5 (7.94) | 6 (9.84) | 11 (16.92) |  |  |
| Prior Chemotherapy History |  |  |  |  | - | 0.541 |
| No | 186 (98.41) | 63 (100.00) | 60 (98.36) | 63 (96.92) |  |  |
| Yes | 3 (1.59) | 0 (0.00) | 1 (1.64) | 2 (3.08) |  |  |
| Clinical Stage |  |  |  |  | 1.42 | 0.842 |
| Stage Ⅰ | 49 (25.93) | 14 (22.22) | 17 (27.87) | 18 (27.69) |  |  |
| Stage Ⅱ | 109 (57.67) | 40 (63.49) | 34 (55.74) | 35 (53.85) |  |  |
| Stage Ⅲ | 31 (16.40) | 9 (14.29) | 10 (16.39) | 12 (18.46) |  |  |
| Lymph Node Metastasis |  |  |  |  | 0.72 | 0.699 |
| No | 113 (59.79) | 38 (60.32) | 34 (55.74) | 41 (63.08) |  |  |
| Yes | 76 (40.21) | 25 (39.68) | 27 (44.26) | 24 (36.92) |  |  |
| Chemotherapy Regimen |  |  |  |  | 6.36 | 0.174 |
| Low emetic risk regimen | 39 (20.63) | 14 (22.22) | 15 (24.59) | 10 (15.38) |  |  |
| Moderate emetic risk regimen | 62 (32.80) | 18 (28.57) | 25 (40.98) | 19 (29.23) |  |  |
| High emetic risk regimen | 88(46.56) | 31 (49.21) | 21 (34.43) | 36(55.38) |  |  |
| Antiemetic Agent |  |  |  |  | 28.80 | **<.001** |
| 5-HT₃ receptor antagonist | 65 (34.39) | 13 (20.63) | 13 (21.31) | 39 (60.00) |  |  |
| 5-HT₃ + NK-1 receptor antagonist | 124 (65.61) | 50 (79.37) | 48 (78.69) | 26 (40.00) |  |  |
| BMI Category |  |  |  |  | - | 0.665 |
| BMI＜18.5 | 6 (3.17) | 3 (4.76) | 2 (3.28) | 1 (1.54) |  |  |
| 18.5≤BMI＜24.0 | 95 (50.26) | 27 (42.86) | 34 (55.74) | 34 (52.31) |  |  |
| 24.0≤BMI＜28.0 | 63 (33.33) | 24 (38.10) | 16 (26.23) | 23 (35.38) |  |  |
| BMI≥28.0 | 25 (13.23) | 9 (14.29) | 9 (14.75) | 7 (10.77) |  |  |
| Surgical Status |  |  |  |  | 1.76 | 0.414 |
| No | 91 (48.15) | 33 (52.38) | 31 (50.82) | 27 (41.54) |  |  |
| Yes | 98 (51.85) | 30 (47.62) | 30 (49.18) | 38 (58.46) |  |  |
| Number of Chemotherapy Cycles |  |  |  |  | 0.70 | 0.705 |
| Four cycles | 47（24.87） | 16（25.40） | 13（21.31） | 18（27.69） |  |  |
| Six cycles | 142（75.13 | 47（74.60） | 48（78.69） | 47（72.31） |  |  |
| M (Q₁, Q₃) | | | | | | |
| Baseline PSQI | 6.00（4.00，9.00） | 6.00（3.50，9.00） | 6.00（4.00，9.00） | 5.00（4.00，8.00） | 0.35# | 0.838 |
| Baseline HADS-D | 5.00（2.00，7.00） | 5.00（2.00，7.00） | 5.00（2.00，7.00） | 4.00（2.00，6.00） | 1.57# | 0.457 |
| Baseline HADS-A | 4.00（2.00，7.00） | 5.00（3.00，7.50） | 4.00（2.00，6.00） | 4.00（2.00，6.00） | 2.79# | 0.248 |
| Baseline FACT-B | 107.00（95.00，116.00） | 101.00（92.00，114.50） | 105.00（97.00，118.00） | 109.00（97.00，116.00） | 2.15# | 0.431 |

Note：#: Kruskal-waills test, χ²: Chi-square test, -: Fisher exact, M: Median, Q₁: 1st Quartile, Q₃: 3st Quartile
