# Supplementary material for: Efficacy and safety of transcutaneous electrical acupoint stimulation and acupressure in alleviating chemotherapy-related adverse reactions in female patients with breast cancer: a randomized clinical trial
Source: Front Oncol. 2026 Apr 15;16:1788635. doi: 10.3389/fonc.2026.1788635 (PMC13124625; doi:10.3389/fonc.2026.1788635)
Supplement: Supplementary file 4 [file Table4.docx]

Analysis results of the ITT dataset

## 1. Acute phase

### （1）Generalized Estimating Equation analysis results (CINV)

| **Variable** | **β (Estimate)** | **Std. Error** | **Wald χ²** | **OR (95% CI)** |
| --- | --- | --- | --- | --- |
| **Acupressure vs. Control** | -0.572 | 0.454 | -1.260 | 0.564 (0.231, 1.377) |
| **TEAS vs. Control** | 0.046 | 0.390 | 0.119 | 1.047 (0.487, 2.251) |
| **cycle2vs.1** | -0.074 | 0.316 | -0.234 | 0.929 (0.500, 1.722) |
| **cycle3vs.1** | 0.242 | 0.312 | 0.776 | 1.274 (0.691, 2.351) |
| **cycle4vs.1** | 0.298 | 0.374 | 0.797 | 1.347 (0.645, 2.813) |
| **5-HT₃ + NK-1 receptor antagonist vs.** **5-HT₃ receptor antagonist** | 0.266 | 0.396 | 0.671 | 1.305 (0.599, 2.842) |
| **Commercial/service industry practitioner vs.** **Worker/Farmer** | -0.074 | 0.360 | -0.206 | 0.929 (0.458, 1.885) |
| **Staff of public institutions/Civil servant vs.** **Worker/Farmer** | -0.020 | 0.398 | -0.051 | 0.980 (0.449, 2.137) |
| **Freelancer vs.** **Worker/Farmer** | 0.422 | 0.398 | 1.059 | 1.525 (0.700, 3.327) |
| **Others vs.** **Worker/Farmer** | -0.073 | 0.261 | -0.281 | 0.930 (0.557, 1.546) |
| **Acupressure*cycle2** | 0.369 | 0.442 | 0.835 | 1.446 (0.607, 3.443) |
| **TEAS *cycle2** | -0.206 | 0.462 | -0.445 | 0.814 (0.329, 2.015) |
| **Acupressure *cycle3** | 0.433 | 0.497 | 0.872 | 1.542 (0.581, 4.109) |
| **TEAS *cycle3** | -0.810 | 0.477 | -1.698 | 0.445 (0.175, 1.130) |
| **Acupressure *cycle4** | -0.689 | 0.571 | -1.208 | 0.502 (0.165, 1.528) |
| **TEAS *cycle4** | -0.613 | 0.506 | -1.211 | 0.542 (0.201, 1.463) |

### （2）Post-event comparison (CINV, interaction not significant)

### （3）The results of the generalized estimating equation analysis (for the degree of nausea)

| **Variable** | **β (Estimate)** | **Std. Error** | **Wald χ²** | **β (95% CI)** |
| --- | --- | --- | --- | --- |
| **Acupressure vs. Control** | -0.418 | 0.527 | -0.794 | -0.418 (-1.455, 0.619) |
| **TEAS vs. Control** | -0.101 | 0.506 | -0.199 | -0.101 (-1.095, 0.893) |
| **cycle2vs.1** | -0.259 | 0.389 | -0.666 | -0.259 (-1.022, 0.504) |
| **cycle3vs.1** | 0.138 | 0.398 | 0.348 | 0.138 (-0.646, 0.922) |
| **cycle4vs.1** | 0.455 | 0.503 | 0.906 | 0.455 (-0.531, 1.441) |
| **5-HT₃ + NK-1 receptor antagonist vs.** **5-HT₃ receptor antagonist** | 0.156 | 0.489 | 0.319 | 0.156 (-0.805, 1.117) |
| **Commercial/service industry practitioner vs.** **Worker/Farmer** | -0.117 | 0.423 | -0.276 | -0.117 (-0.948, 0.714) |
| **Staff of public institutions/Civil servant vs.** **Worker/Farmer** | 0.072 | 0.455 | 0.157 | 0.072 (-0.823, 0.967) |
| **Freelancer vs.** **Worker/Farmer** | 0.875 | 0.586 | 1.493 | 0.875 (-0.274, 2.024) |
| **Others vs.** **Worker/Farmer** | -0.389 | 0.348 | -1.116 | -0.389 (-1.074, 0.296) |
| **Acupressure*cycle2** | 0.294 | 0.527 | 0.557 | 0.294 (-0.741, 1.329) |
| **TEAS *cycle2** | -0.332 | 0.550 | -0.603 | -0.332 (-1.407, 0.743) |
| **Acupressure *cycle3** | 0.403 | 0.604 | 0.667 | 0.403 (-0.785, 1.591) |
| **TEAS *cycle3** | -0.602 | 0.544 | -1.108 | -0.602 (-1.668, 0.464) |
| **Acupressure *cycle4** | -0.724 | 0.628 | -1.153 | -0.724 (-1.956, 0.508) |
| **TEAS *cycle4** | -0.728 | 0.630 | -1.154 | -0.728 (-1.961, 0.505) |

### （4）Post-event comparison (severe nausea, no significant interaction)

## 2. Delayed period

### （1）Generalized Estimating Equation analysis results (CINV)

| **Variable** | **β (Estimate)** | **Std. Error** | **Wald χ²** | **OR(95% CI)** |
| --- | --- | --- | --- | --- |
| **Acupressure vs. Control** | -0.367 | 0.384 | 0.955 | 0.693 (0.326, 1.471) |
| **TEAS vs. Control** | -0.549 | 0.366 | 1.498 | 0.578 (0.282, 1.183) |
| **cycle2vs.1** | 0.137 | 0.234 | 0.586 | 1.147 (0.725, 1.814) |
| **cycle3vs.1** | 0.626 | 0.286 | 2.188 | 1.870 (1.067, 3.278)* |
| **cycle4vs.1** | 0.511 | 0.291 | 1.755 | 1.667 (0.942, 2.950) |
| **5-HT₃ + NK-1 receptor antagonist vs.** **5-HT₃ receptor antagonist** | 0.325 | 0.390 | 0.834 | 0.840 (0.533, 1.326) |
| **Commercial/service industry practitioner vs.** **Worker/Farmer** | 0.258 | 0.366 | 0.705 | 1.384 (0.645, 2.970) |
| **Staff of public institutions/Civil servant vs.** **Worker/Farmer** | 0.170 | 0.377 | 0.452 | 1.295 (0.631, 2.654) |
| **Freelancer vs.** **Worker/Farmer** | 0.082 | 0.387 | 0.212 | 1.185 (0.566, 2.482) |
| **Others vs.** **Worker/Farmer** | -0.174 | 0.233 | 0.749 | 1.086 (0.508, 2.320) |
| **Acupressure*cycle2** | 0.150 | 0.359 | 0.418 | 1.162 (0.575, 2.349) |
| **TEAS *cycle2** | 0.219 | 0.357 | 0.612 | 1.245 (0.618, 2.506) |
| **Acupressure *cycle3** | 0.176 | 0.427 | 0.414 | 1.193 (0.516, 2.754) |
| **TEAS *cycle3** | -0.669 | 0.436 | 1.533 | 0.512 (0.218, 1.205) |
| **Acupressure *cycle4** | -0.468 | 0.445 | 1.051 | 0.626 (0.262, 1.497) |
| **TEAS *cycle4** | -0.858 | 0.430 | 1.996 | 0.424 (0.183, 0.985)* |

### （2）Post-event comparison (CINV)

| **Number of chemotherapy cycles** | **Comparison** | **OR (95% CI)** |
| --- | --- | --- |
| **cycle1** | Acupressure vs Control | 0.694 (0.275, 1.742) |
|  | TEAS vs Control | 0.578 (0.240, 1.391) |
|  | TEAS vs Acupressure | 0.833 (0.324, 2.145) |
| **cycle2** | Acupressure vs Control | 0.806 (0.323, 2.008) |
|  | TEAS vs Control | 0.719 (0.297, 1.745) |
|  | TEAS vs Acupressure | 0.893 (0.361, 2.207) |
| **cycle3** | Acupressure vs Control | 0.826 (0.339, 2.016) |
|  | TEAS vs Control | 0.296 (0.115, 0.764)* |
|  | TEAS vs Acupressure | 0.359 (0.138, 0.929)* |
| **cycle4** | Acupressure vs Control | 0.435 (0.177, 1.063) |
|  | TEAS vs Control | 0.245 (0.095, 0.635)* |
|  | TEAS vs Acupressure | 0.565 (0.208, 1.529) |

### （3）The results of the generalized estimating equation analysis (for the degree of nausea)

| **Variable** | **β (Estimate)** | **Std. Error** | **Wald χ²** | **β (95% CI)** |
| --- | --- | --- | --- | --- |
| **Acupressure vs. Control** | -0.602 | 0.530 | 1.135 | -0.602 (-1.641, 0.437) |
| **TEAS vs. Control** | -0.290 | 0.523 | 0.555 | -0.290 (-1.315, 0.735) |
| **cycle2vs.1** | 0.238 | 0.359 | 0.664 | 0.238 (-0.465, 0.941) |
| **cycle3vs.1** | 0.732 | 0.441 | 1.662 | 0.732 (-0.132, 1.596) |
| **cycle4vs.1** | 0.908 | 0.489 | 1.855 | 0.908 (-0.051, 1.867) |
| **5-HT₃ + NK-1 receptor antagonist vs.** **5-HT₃ receptor antagonist** | -0.490 | 0.309 | 1.587 | -0.490 (-1.095, 0.115) |
| **Commercial/service industry practitioner vs.** **Worker/Farmer** | 0.306 | 0.566 | 0.541 | 0.306 (-0.803, 1.415) |
| **Staff of public institutions/Civil servant vs.** **Worker/Farmer** | 0.079 | 0.524 | 0.151 | 0.079 (-0.949, 1.107) |
| **Freelancer vs.** **Worker/Farmer** | -0.069 | 0.507 | 0.135 | -0.069 (-1.063, 0.925) |
| **Others vs.** **Worker/Farmer** | 0.230 | 0.614 | 0.374 | 0.230 (-0.973, 1.433) |
| **Acupressure*cycle2** | 0.434 | 0.496 | 0.875 | 0.434 (-0.539, 1.407) |
| **TEAS *cycle2** | 0.023 | 0.484 | 0.048 | 0.023 (-0.926, 0.972) |
| **Acupressure *cycle3** | 0.780 | 0.591 | 1.320 | 0.780 (-0.379, 1.939) |
| **TEAS *cycle3** | -0.730 | 0.589 | 1.239 | -0.730 (-1.884, 0.424) |
| **Acupressure *cycle4** | -0.823 | 0.612 | 1.344 | -0.823 (-2.022, 0.376) |
| **TEAS *cycle4** | -1.256 | 0.617 | 2.036 | -1.256 (-2.465, -0.047)* |

### （4）Comparison after the event (in terms of nausea level)

| **Number of chemotherapy cycles** | **Comparison** | **β (95% CI)** |
| --- | --- | --- |
| **cycle1** | Acupressure vs Control | 0.602 (-0.670, 1.874) |
|  | TEAS vs Control | 0.290 (-0.964, 1.545) |
|  | TEAS vs Acupressure | -0.311 (-1.509, 0.886) |
| **cycle2** | Acupressure vs Control | -0.168 (-1.150, 0.814) |
|  | TEAS vs Control | -0.267 (-1.557, 1.023) |
|  | TEAS vs Acupressure | -0.100 (-1.340, 1.140) |
| **cycle3** | Acupressure vs Control | 0.178 (-1.114, 1.470) |
|  | TEAS vs Control | -1.020 (-2.312, 0.271) |
|  | TEAS vs Acupressure | -1.199 (-2.465, 0.068) |
| **cycle4** | Acupressure vs Control | -1.425 (-2.725, -0.125)* |
|  | TEAS vs Control | -1.547 (-2.904, -0.189)* |
|  | TEAS vs Acupressure | -0.122 (-1.320, 1.076) |

## 3. Sleep quality

### （1）The results of generalized estimating equation analysis

| **Variable** | **β (Estimate)** | **Std. Error** | **Wald χ²** | **β (95% CI)** |
| --- | --- | --- | --- | --- |
| **Acupressure vs. Control** | 0.194 | 0.752 | 0.258 | 0.194 (-1.281, 1.669) |
| **TEAS vs. Control** | -0.470 | 0.678 | -0.694 | -0.470 (-1.799, 0.859) |
| **cycle2vs.1** | 0.313 | 0.565 | 0.555 | 0.313 (-0.794, 1.420) |
| **cycle3vs.1** | 0.440 | 0.600 | 0.733 | 0.440 (-0.737, 1.617) |
| **cycle4vs.1** | 0.554 | 0.651 | 0.851 | 0.554 (-0.721, 1.829) |
| **5-HT₃ + NK-1 receptor antagonist vs.** **5-HT₃ receptor antagonist** | -0.119 | 0.393 | -0.304 | -0.119 (-0.889, 0.651) |
| **Commercial/service industry practitioner vs.** **Worker/Farmer** | -0.985 | 0.729 | -1.352 | -0.985 (-2.414, 0.444) |
| **Staff of public institutions/Civil servant vs.** **Worker/Farmer** | 0.117 | 0.645 | 0.181 | 0.117 (-1.148, 1.382) |
| **Freelancer vs.** **Worker/Farmer** | 0.465 | 0.826 | 0.563 | 0.465 (-1.154, 2.084) |
| **Others vs.** **Worker/Farmer** | 0.527 | 0.830 | 0.635 | 0.527 (-1.100, 2.154) |
| **Acupressure*cycle2** | -1.127 | 0.733 | -1.537 | -1.127 (-2.564, 0.310) |
| **TEAS *cycle2** | -0.688 | 0.714 | -0.963 | -0.688 (-2.088, 0.712) |
| **Acupressure *cycle3** | -1.052 | 0.847 | -1.241 | -1.052 (-2.712, 0.608) |
| **TEAS *cycle3** | -1.581 | 0.796 | -1.988 | -1.581 (-3.141, -0.021)* |
| **Acupressure *cycle4** | -0.858 | 0.847 | -1.013 | -0.858 (-2.518, 0.802) |
| **TEAS *cycle4** | -1.595 | 0.826 | -1.932 | -1.595 (-3.214, 0.024) |

### （2）Post-event comparison

| **Number of chemotherapy cycles** | **Comparison** | **β (95% CI)** |
| --- | --- | --- |
| **cycle1** | Acupressure vs Control | 0.150 (-1.670, 1.969) |
|  | TEAS vs Control | -0.465 (-2.090, 1.164) |
|  | TEAS vs Acupressure | -0.615 (-2.350, 1.123) |
| **cycle2** | Acupressure vs Control | -0.926 (-2.490, 0.639) |
|  | TEAS vs Control | -1.162 (-2.790, 0.467) |
|  | TEAS vs Acupressure | -0.237 (-1.880, 1.404) |
| **cycle3** | Acupressure vs Control | -1.017 (-2.920, 0.886) |
|  | TEAS vs Control | -2.008 (-3.800, -0.217) |
|  | TEAS vs Acupressure | -0.991 (-2.670, 0.685) |
| **cycle4** | Acupressure vs Control | -0.671 (-2.300, 0.957) |
|  | TEAS vs Control | -2.078 (-3.740, -0.417) |
|  | TEAS vs Acupressure | -1.407 (-3.020, 0.203) |

## 4. Anxiety

### （1）The results of generalized estimating equation analysis

| **Variable** | **β (Estimate)** | **Std. Error** | **Wald χ²** | **β (95% CI)** |
| --- | --- | --- | --- | --- |
| **Acupressure vs. Control** | -1.382 | 0.556 | -2.486 | -1.382 (-2.473, -0.291)* |
| **TEAS vs. Control** | 0.300 | 0.613 | 0.490 | 0.300 (-0.902, 1.502) |
| **cycle2vs.1** | -0.618 | 0.353 | -1.753 | -0.618 (-1.310, 0.074) |
| **cycle3vs.1** | -0.969 | 0.421 | -2.303 | -0.969 (-1.796, -0.142)* |
| **cycle4vs.1** | -0.200 | 0.469 | -0.427 | -0.200 (-1.117, 0.717) |
| **5-HT₃ + NK-1 receptor antagonist vs.** **5-HT₃ receptor antagonist** | 0.729 | 0.349 | 2.090 | 0.729 (0.045, 1.413)* |
| **Commercial/service industry practitioner vs.** **Worker/Farmer** | -0.571 | 0.580 | -0.984 | -0.571 (-1.712, 0.570) |
| **Staff of public institutions/Civil servant vs.** **Worker/Farmer** | -0.105 | 0.503 | -0.208 | -0.105 (-1.092, 0.882) |
| **Freelancer vs.** **Worker/Farmer** | -0.948 | 0.552 | -1.716 | -0.948 (-2.033, 0.137) |
| **Others vs.** **Worker/Farmer** | 0.488 | 0.636 | 0.767 | 0.488 (-0.764, 1.740) |
| **Acupressure*cycle2** | 0.644 | 0.532 | 1.211 | 0.644 (-0.399, 1.687) |
| **TEAS *cycle2** | -0.274 | 0.459 | -0.598 | -0.274 (-1.174, 0.626) |
| **Acupressure *cycle3** | 1.051 | 0.612 | 1.719 | 1.051 (-0.151, 2.253) |
| **TEAS *cycle3** | 0.069 | 0.561 | 0.123 | 0.069 (-1.035, 1.173) |
| **Acupressure *cycle4** | -0.348 | 0.632 | -0.551 | -0.348 (-1.588, 0.892) |
| **TEAS *cycle4** | -1.217 | 0.611 | -1.993 | -1.217 (-2.416, -0.018)* |

### （2）Post-event comparison

| **Number of chemotherapy cycles** | **Comparison** | **β (95% CI)** |
| --- | --- | --- |
| **cycle1** | Acupressure vs Control | -1.382(-2.716, -0.048)* |
|  | TEAS vs Control | 0.300 (-1.770, 1.170) |
|  | TEAS vs Acupressure | 1.682 (0.170, 3.194)* |
| **cycle2** | Acupressure vs Control | -0.738 (-1.825, 0.349) |
|  | TEAS vs Control | 0.026 (-1.385, 1.333) |
|  | TEAS vs Acupressure | 0.764 (-0.498, 2.026) |
| **cycle3** | Acupressure vs Control | -0.331 (-1.559, 0.897) |
|  | TEAS vs Control | 0.369 (-1.081, 1.820) |
|  | TEAS vs Acupressure | 0.700 (-0.760, 2.161) |
| **cycle4** | Acupressure vs Control | -1.730 (0.591, 2.869)* |
|  | TEAS vs Control | -0.917 (-2.306, 0.472) |
|  | TEAS vs Acupressure | -0.813 (-2.056, 0.430) |

## 5. Depression

### （1）The results of generalized estimating equation analysis

| **Variable** | **β (Estimate)** | **Std. Error** | **Wald χ²** | **β (95% CI)** |
| --- | --- | --- | --- | --- |
| **Acupressure vs. Control** | -0.980 | 0.488 | -2.010 | -0.980 (-1.932, -0.028)* |
| **TEAS vs. Control** | 0.411 | 0.545 | 0.753 | 0.411 (-0.663, 1.485) |
| **cycle2vs.1** | -0.048 | 0.346 | -0.140 | -0.048 (-0.722, 0.626) |
| **cycle3vs.1** | -0.209 | 0.362 | -0.578 | -0.209 (-0.919, 0.501) |
| **cycle4vs.1** | 0.289 | 0.393 | 0.737 | 0.289 (-0.480, 1.058) |
| **5-HT₃ + NK-1 receptor antagonist vs.** **5-HT₃ receptor antagonist** | 0.050 | 0.259 | 0.195 | 0.050 (-0.459, 0.559) |
| **Commercial/service industry practitioner vs.** **Worker/Farmer** | 0.074 | 0.523 | 0.141 | 0.074 (-0.952, 1.099) |
| **Staff of public institutions/Civil servant vs.** **Worker/Farmer** | 0.855 | 0.447 | 1.913 | 0.855 (-0.021, 1.731) |
| **Freelancer vs.** **Worker/Farmer** | -0.060 | 0.568 | -0.107 | -0.060 (-1.177, 1.057) |
| **Others vs.** **Worker/Farmer** | 0.871 | 0.581 | 1.499 | 0.871 (-0.264, 2.006) |
| **Acupressure*cycle2** | 0.359 | 0.474 | 0.757 | 0.359 (-0.571, 1.289) |
| **TEAS *cycle2** | -0.607 | 0.482 | -1.259 | -0.607 (-1.552, 0.338) |
| **Acupressure *cycle3** | 0.390 | 0.527 | 0.740 | 0.390 (-0.647, 1.427) |
| **TEAS *cycle3** | -0.220 | 0.526 | -0.417 | -0.220 (-1.254, 0.814) |
| **Acupressure *cycle4** | -0.272 | 0.540 | -0.505 | -0.272 (-1.331, 0.787) |
| **TEAS *cycle4** | -1.305 | 0.561 | -2.326 | -1.305 (-2.405, -0.205)* |

### （2）Post-event comparison

| **Number of chemotherapy cycles** | **Comparison** | **β (95% CI)** |
| --- | --- | --- |
| **cycle1** | Acupressure vs Control | -0.980 (-2.152, 0.192) |
|  | TEAS vs Control | 0.411 (-0.897, 1.719) |
|  | TEAS vs Acupressure | 1.391 (0.119, 2.663)* |
| **cycle2** | Acupressure vs Control | -0.621 (-1.600, 0.357) |
|  | TEAS vs Control | -0.197 (-1.421, 1.028) |
|  | TEAS vs Acupressure | -0.425 (-1.555, 0.705) |
| **cycle3** | Acupressure vs Control | -0.591 (-1.662, 0.480) |
|  | TEAS vs Control | 0.191 (-1.057, 1.439) |
|  | TEAS vs Acupressure | 0.782 (-0.503, 2.066) |
| **cycle4** | Acupressure vs Control | -1.253 (-2.223, -0.282)* |
|  | TEAS vs Control | -0.894 (-2.042, 0.254) |
|  | TEAS vs Acupressure | 0.359 (-0.703, 1.421) |

## 6. Quality of life

### （1）The results of generalized estimating equation analysis

| **Variable** | **β (Estimate)** | **Std. Error** | **Wald χ²** | **β (95% CI)** |
| --- | --- | --- | --- | --- |
| **Acupressure vs. Control** | 1.251 | 2.383 | 0.525 | 1.251 (-3.420, 5.922) |
| **TEAS vs. Control** | 5.286 | 2.155 | 2.453 | 5.286 (1.062, 9.510)* |
| **cycle2vs.1** | -2.128 | 1.556 | -1.368 | -2.128 (-5.168, 0.912) |
| **cycle3vs.1** | -0.905 | 1.611 | -0.562 | -0.905 (-4.053, 2.243) |
| **cycle4vs.1** | -3.043 | 1.650 | -1.845 | -3.043 (-6.277, 0.191) |
| **5-HT₃ + NK-1 receptor antagonist vs.** **5-HT₃ receptor antagonist** | 3.781 | 2.189 | 1.727 | 3.781 (-0.500, 8.062) |
| **Commercial/service industry practitioner vs.** **Worker/Farmer** | 0.870 | 1.945 | 0.447 | 0.870 (-2.950, 4.690) |
| **Staff of public institutions/Civil servant vs.** **Worker/Farmer** | 0.913 | 2.057 | 0.444 | 0.913 (-3.122, 4.948) |
| **Freelancer vs.** **Worker/Farmer** | -0.248 | 2.527 | -0.098 | -0.248 (-5.193, 4.697) |
| **Others vs.** **Worker/Farmer** | 0.777 | 1.294 | 0.600 | 0.777 (-1.769, 3.323) |
| **Acupressure*cycle2** | 3.820 | 2.133 | 1.790 | 3.820 (-0.361, 8.001) |
| **TEAS *cycle2** | 4.541 | 1.977 | 2.297 | 4.541 (0.666, 8.416)* |
| **Acupressure *cycle3** | 3.384 | 2.426 | 1.395 | 3.384 (-1.380, 8.148) |
| **TEAS *cycle3** | 4.198 | 2.057 | 2.041 | 4.198 (0.160, 8.236)* |
| **Acupressure *cycle4** | 6.809 | 2.384 | 2.856 | 6.809 (2.131, 11.487)** |
| **TEAS *cycle4** | 7.852 | 2.117 | 3.709 | 7.852 (3.699, 12.005)** |

### （2）Post-event comparison

| **Number of chemotherapy cycles** | **Comparison** | **β (95% CI)** |
| --- | --- | --- |
| **cycle1** | Acupressure vs Control | 1.250 (-4.468, 6.968) |
|  | TEAS vs Control | 5.290 (0.116, 10.464)* |
|  | TEAS vs Acupressure | 4.030 (-1.007, 9.067) |
| **cycle2** | Acupressure vs Control | 5.070 (-0.155, 10.300) |
|  | TEAS vs Control | 9.830 (4.438, 15.210)*** |
|  | TEAS vs Acupressure | 4.760 (-0.430, 9.940) |
| **cycle3** | Acupressure vs Control | 4.630 (-0.350, 9.620) |
|  | TEAS vs Control | 9.480 (4.661, 14.310)*** |
|  | TEAS vs Acupressure | 4.850 (-0.402, 10.100) |
| **cycle4** | Acupressure vs Control | 8.060 (3.404, 12.710)*** |
|  | TEAS vs Control | 13.140 (8.105, 18.170)*** |
|  | TEAS vs Acupressure | 5.080 (0.778, 9.380)* |
