# Supplementary material for: Efficacy and safety of transcutaneous electrical acupoint stimulation and acupressure in alleviating chemotherapy-related adverse reactions in female patients with breast cancer: a randomized clinical trial
Source: Front Oncol. 2026 Apr 15;16:1788635. doi: 10.3389/fonc.2026.1788635 (PMC13124625; doi:10.3389/fonc.2026.1788635)
Supplement: Supplementary file 5 [file Table5.docx]

Analysis results of the PPS dataset

## 1. Acute phase

### （1）Generalized Estimating Equation analysis results (CINV)

| **Variable** | **β (Estimate)** | **Std. Error** | **Wald χ²** | **OR (95% CI)** |
| --- | --- | --- | --- | --- |
| **Acupressure vs. Control** | -0.590 | 0.463 | 1.623 | 0.555 (0.224-1.375) |
| **TEAS vs. Control** | 0.114 | 0.398 | 0.080 | 1.121 (0.514-2.445) |
| **cycle2vs.1** | -0.074 | 0.331 | 0.051 | 0.928 (0.485-1.778) |
| **cycle3vs.1** | 0.294 | 0.315 | 0.883 | 1.342 (0.725-2.486) |
| **cycle4vs.1** | 0.297 | 0.378 | 0.622 | 1.346 (0.641-2.825) |
| **5-HT₃ + NK-1 receptor antagonist vs.** **5-HT₃ receptor antagonist** | -0.138 | 0.258 | 0.293 | 0.871 (0.526-1.444) |
| **Commercial/service industry practitioner vs.** **Worker/Farmer** | 0.337 | 0.424 | 0.632 | 1.401 (0.610-3.220) |
| **Staff of public institutions/Civil servant vs.** **Worker/Farmer** | 0.026 | 0.372 | 0.001 | 1.026 (0.495-2.127) |
| **Freelancer vs.** **Worker/Farmer** | 0.097 | 0.405 | 0.061 | 1.102 (0.498-2.437) |
| **Others vs.** **Worker/Farmer** | 0.539 | 0.412 | 1.721 | 1.715 (0.766-3.842) |
| **Acupressure*cycle2** | 0.387 | 0.451 | 0.743 | 1.473 (0.609-3.563) |
| **TEAS *cycle2** | -0.421 | 0.477 | 0.782 | 0.657 (0.258-1.671) |
| **Acupressure *cycle3** | 0.441 | 0.497 | 0.790 | 1.554 (0.586-4.118) |
| **TEAS *cycle3** | -0.992 | 0.476 | 4.340 | 0.371 (0.146-0.943) |
| **Acupressure *cycle4** | -0.673 | 0.568 | 1.413 | 0.510 (0.168-1.551) |
| **TEAS *cycle4** | -0.709 | 0.513 | 1.911 | 0.492 (0.180-1.346) |

### （2）Post-event comparison (CINV)

| **Number of chemotherapy cycles** | **comparison** | **OR (95% CI)** |
| --- | --- | --- |
| **cycle1** | Acupressure vs Control | 0.556 (0.182, 1.686) |
|  | TEAS vs Control | 1.124 (0.431, 2.934) |
|  | TEAS vs Acupressure | 2.041 (0.685, 6.085) |
| **cycle2** | Acupressure vs Control | 0.820 (0.283, 2.374) |
|  | TEAS vs Control | 0.735 (0.256, 2.109) |
|  | TEAS vs Acupressure | 0.901 (0.318, 2.551) |
| **cycle3** | Acupressure vs Control | 0.862 (0.334, 2.224) |
|  | TEAS vs Control | 0.415 (0.149, 1.157) |
|  | TEAS vs Acupressure | 0.483 (0.166, 1.404) |
| **cycle4** | Acupressure vs Control | 0.282 (0.088, 0.913)* |
|  | TEAS vs Control | 0.552 (0.200, 1.515) |
|  | TEAS vs Acupressure | 1.961 (0.556, 6.918) |

### （3）The results of the generalized estimating equation analysis (for the degree of nausea)

| **Variable** | **β (Estimate)** | **Std. Error** | **Wald χ²** | **β (95% CI)** |
| --- | --- | --- | --- | --- |
| **Acupressure vs. Control** | -0.443 | 0.531 | 0.690 | (-1.484, 0.598) |
| **TEAS vs. Control** | -0.038 | 0.522 | 0.010 | (-1.061, 0.985) |
| **cycle2vs.1** | -0.247 | 0.401 | 0.380 | (-1.033, 0.539) |
| **cycle3vs.1** | 0.175 | 0.404 | 0.190 | (-0.617, 0.967) |
| **cycle4vs.1** | 0.429 | 0.498 | 0.740 | (-0.547, 1.405) |
| **5-HT₃ + NK-1 receptor antagonist vs.** **5-HT₃ receptor antagonist** | -0.477 | 0.351 | 1.850 | (-1.165, 0.211) |
| **Commercial/service industry practitioner vs.** **Worker/Farmer** | 0.249 | 0.508 | 0.240 | (-0.747, 1.245) |
| **Staff of public institutions/Civil servant vs.** **Worker/Farmer** | -0.033 | 0.427 | 0.010 | (-0.870, 0.804) |
| **Freelancer vs.** **Worker/Farmer** | 0.147 | 0.458 | 0.100 | (-0.751, 1.045) |
| **Others vs.** **Worker/Farmer** | 0.992 | 0.593 | 2.800 | (-0.170, 2.154) |
| **Acupressure*cycle2** | 0.287 | 0.536 | 0.290 | (-0.764, 1.338) |
| **TEAS *cycle2** | -0.574 | 0.549 | 1.090 | (-1.650, 0.502) |
| **Acupressure *cycle3** | 0.414 | 0.601 | 0.470 | (-0.764, 1.592) |
| **TEAS *cycle3** | -0.780 | 0.533 | 2.140 | (-1.825, 0.265) |
| **Acupressure *cycle4** | -0.700 | 0.621 | 1.270 | (-1.917, 0.517) |
| **TEAS *cycle4** | -0.857 | 0.633 | 1.840 | (-2.098, 0.384) |

### （4）Post-event comparison (severe nausea level, interaction term not significant)

## 2. Delayed period

### （1）Generalized Estimating Equation analysis results (CINV)

| **Variable** | **β (Estimate)** | **Std. Error** | **Wald χ²** | **OR(95% CI)** |
| --- | --- | --- | --- | --- |
| **Acupressure vs. Control** | -0.344 | 0.388 | 0.787 | 0.709 (0.331, 1.517) |
| **TEAS vs. Control** | -0.547 | 0.377 | 2.11 | 0.579 (0.277, 1.210) |
| **cycle2vs.1** | 0.078 | 0.234 | 0.111 | 1.081 (0.684, 1.708) |
| **cycle3vs.1** | 0.580 | 0.289 | 4.028 | 1.785 (1.013, 3.147)* |
| **cycle4vs.1** | 0.514 | 0.295 | 3.038 | 1.672 (0.938, 2.981) |
| **5-HT₃ + NK-1 receptor antagonist vs.** **5-HT₃ receptor antagonist** | -0.202 | 0.235 | 0.741 | 0.817 (0.516, 1.294) |
| **Commercial/service industry practitioner vs.** **Worker/Farmer** | 0.291 | 0.402 | 0.524 | 1.338 (0.609, 2.941) |
| **Staff of public institutions/Civil servant vs.** **Worker/Farmer** | 0.325 | 0.374 | 0.756 | 1.385 (0.665, 2.883) |
| **Freelancer vs.** **Worker/Farmer** | 0.227 | 0.387 | 0.344 | 1.255 (0.588, 2.679) |
| **Others vs.** **Worker/Farmer** | 0.147 | 0.395 | 0.139 | 1.159 (0.534, 2.514) |
| **Acupressure*cycle2** | 0.204 | 0.359 | 0.322 | 1.226 (0.607, 2.477) |
| **TEAS *cycle2** | 0.245 | 0.372 | 0.434 | 1.277 (0.616, 2.648) |
| **Acupressure *cycle3** | 0.228 | 0.430 | 0.280 | 1.256 (0.540, 2.919) |
| **TEAS *cycle3** | -0.672 | 0.446 | 2.267 | 0.511 (0.213, 1.225) |
| **Acupressure *cycle4** | -0.438 | 0.448 | 0.955 | 0.645 (0.268, 1.552) |
| **TEAS *cycle4** | -0.998 | 0.440 | 5.155 | 0.369 (0.156, 0.874)* |

### （2）Post-event comparison (CINV)

| **Number of chemotherapy cycles** | **comparison** | **OR (95% CI)** |
| --- | --- | --- |
| **cycle1** | Acupressure vs Control | 0.709 (0.331, 1.517) |
|  | TEAS vs Control | 0.579 (0.277, 1.210) |
|  | TEAS vs Acupressure | 0.816 (0.391, 1.705) |
| **cycle2** | Acupressure vs Control | 0.870 (0.458, 1.653) |
|  | TEAS vs Control | 0.741 (0.391, 1.405) |
|  | TEAS vs Acupressure | 0.852 (0.451, 1.609) |
| **cycle3** | Acupressure vs Control | 0.893 (0.447, 1.784) |
|  | TEAS vs Control | 0.296 (0.115, 0.761)* |
|  | TEAS vs Acupressure | 0.331 (0.135, 0.814)* |
| **cycle4** | Acupressure vs Control | 0.459 (0.185, 1.132) |
|  | TEAS vs Control | 0.213 (0.080, 0.566)* |
|  | TEAS vs Acupressure | 0.465 (0.181, 1.192) |

### （3）The results of the generalized estimating equation analysis (for the degree of nausea)

| **Variable** | **β (Estimate)** | **Std. Error** | **Wald χ²** | **β (95% CI)** |
| --- | --- | --- | --- | --- |
| **Acupressure vs. Control** | -0.572 | 0.535 | 1.145 | -0.572 (-1.621, 0.477) |
| **TEAS vs. Control** | -0.238 | 0.539 | 0.190 | -0.238 (-1.294, 0.818) |
| **cycle2vs.1** | 0.120 | 0.357 | 0.110 | 0.120 (-0.580, 0.820) |
| **cycle3vs.1** | 0.667 | 0.449 | 2.210 | 0.667 (-0.213, 1.547) |
| **cycle4vs.1** | 0.921 | 0.497 | 3.443 | 0.921 (-0.053, 1.895) |
| **5-HT₃ + NK-1 receptor antagonist vs.** **5-HT₃ receptor antagonist** | -0.528 | 0.314 | 2.832 | -0.528 (-1.143, 0.087) |
| **Commercial/service industry practitioner vs.** **Worker/Farmer** | 0.260 | 0.589 | 0.233 | 0.260 (-0.895, 1.415) |
| **Staff of public institutions/Civil servant vs.** **Worker/Farmer** | 0.158 | 0.542 | 0.092 | 0.158 (-0.905, 1.221) |
| **Freelancer vs.** **Worker/Farmer** | 0.040 | 0.527 | 0.012 | 0.040 (-0.993, 1.073) |
| **Others vs.** **Worker/Farmer** | 0.311 | 0.628 | 0.243 | 0.311 (-0.920, 1.542) |
| **Acupressure*cycle2** | 0.545 | 0.493 | 1.222 | 0.545 (-0.422, 1.512) |
| **TEAS *cycle2** | 0.054 | 0.49 | 0.011 | 0.054 (-0.906, 1.014) |
| **Acupressure *cycle3** | 0.860 | 0.601 | 2.054 | 0.860 (-0.318, 2.038) |
| **TEAS *cycle3** | -0.708 | 0.602 | 1.383 | -0.708 (-1.888, 0.472) |
| **Acupressure *cycle4** | -0.796 | 0.616 | 1.672 | -0.796 (-2.003, 0.411) |
| **TEAS *cycle4** | -1.492 | 0.620 | 5.791 | -1.492 (-2.707, -0.277)* |

### （4）Comparison after the event (in terms of nausea level)

| **Number of chemotherapy cycles** | **comparison** | **β (95% CI)** |
| --- | --- | --- |
| **cycle1** | Acupressure vs Control | -0.572 (-1.856, 0.713) |
|  | TEAS vs Control | -0.237 (-1.530, 1.055) |
|  | TEAS vs Acupressure | 0.334 (-0.902, 1.570) |
| **cycle2** | Acupressure vs Control | -0.027 (-1.342, 1.289) |
|  | TEAS vs Control | -0.183 (-1.501, 1.135) |
|  | TEAS vs Acupressure | -0.156 (-1.433, 1.121) |
| **cycle3** | Acupressure vs Control | 0.288 (-1.025, 1.601) |
|  | TEAS vs Control | -0.946 (-2.262, 0.371) |
|  | TEAS vs Acupressure | -1.234 (-2.535, 0.068) |
| **cycle4** | Acupressure vs Control | -1.368 (-2.698, -0.039)* |
|  | TEAS vs Control | -1.730 (-3.106, -0.354)* |
|  | TEAS vs Acupressure | -0.362 (-1.578, 0.854) |

## 3. Sleep quality

### （1）The results of generalized estimating equation analysis

| **Variable** | **β (Estimate)** | **Std. Error** | **Wald χ²** | **β (95% CI)** |
| --- | --- | --- | --- | --- |
| **Acupressure vs. Control** | 0.299 | 0.750 | 0.160 | 0.299 (-1.171, 1.769) |
| **TEAS vs. Control** | -0.403 | 0.701 | 0.330 | -0.403 (-1.777, 0.971) |
| **cycle2vs.1** | 0.328 | 0.580 | 0.320 | 0.328 (-0.809, 1.465) |
| **cycle3vs.1** | 0.476 | 0.600 | 0.630 | 0.476 (-0.700, 1.652) |
| **cycle4vs.1** | 0.524 | 0.648 | 0.650 | 0.524 (-0.746, 1.794) |
| **5-HT₃ + NK-1 receptor antagonist vs.** **5-HT₃ receptor antagonist** | -0.217 | 0.392 | 0.300 | -0.217 (-0.985, 0.551) |
| **Commercial/service industry practitioner vs.** **Worker/Farmer** | -1.101 | 0.755 | 2.120 | -1.101 (-2.581, 0.379) |
| **Staff of public institutions/Civil servant vs.** **Worker/Farmer** | 0.118 | 0.668 | 0.030 | 0.118 (-1.191, 1.427) |
| **Freelancer vs.** **Worker/Farmer** | 0.448 | 0.859 | 0.270 | 0.448 (-1.235, 2.131) |
| **Others vs.** **Worker/Farmer** | 0.612 | 0.847 | 0.520 | 0.612 (-1.048, 2.272) |
| **Acupressure*cycle2** | -1.120 | 0.750 | 2.230 | -1.120 (-2.590, 0.350) |
| **TEAS *cycle2** | -0.714 | 0.725 | 0.970 | -0.714 (-2.135, 0.707) |
| **Acupressure *cycle3** | -1.085 | 0.852 | 1.620 | -1.085 (-2.755, 0.585) |
| **TEAS *cycle3** | -1.592 | 0.792 | 4.040 | -1.592 (-3.144, -0.040)* |
| **Acupressure *cycle4** | -0.775 | 0.838 | 0.860 | -0.775 (-2.417, 0.867) |
| **TEAS *cycle4** | -1.636 | 0.818 | 4.000 | -1.636 (-3.239, -0.033)* |

### （2）Post-event comparison

| **Number of chemotherapy cycles** | **comparison** | **β (95% CI)** |
| --- | --- | --- |
| **cycle1** | Acupressure vs Control | -0.299 (-2.099, 1.501) |
|  | TEAS vs Control | 0.403 (-1.278, 2.084) |
|  | TEAS vs Acupressure | 0.703 (-1.077, 2.483) |
| **cycle2** | Acupressure vs Control | -0.821 (-2.367, 0.725) |
|  | TEAS vs Control | -1.117 (-2.755, 0.521) |
|  | TEAS vs Acupressure | -0.296 (-1.900, 1.308) |
| **cycle3** | Acupressure vs Control | -0.786 (-2.644, 1.072) |
|  | TEAS vs Control | -1.995 (-3.761, -0.229)* |
|  | TEAS vs Acupressure | -1.210 (-2.800, 0.380) |
| **cycle4** | Acupressure vs Control | -0.476 (-2.119, 1.167) |
|  | TEAS vs Control | -2.039 (-3.635, -0.443)* |
|  | TEAS vs Acupressure | -1.563 (-3.050, -0.076)* |

## 4. Anxiety

### （1）The results of generalized estimating equation analysis

| **Variable** | **β (Estimate)** | **Std. Error** | **Wald χ²** | **β (95% CI)** |
| --- | --- | --- | --- | --- |
| **Acupressure vs. Control** | -1.246 | 0.553 | 5.070 | -1.246 (-2.330, -0.162)* |
| **TEAS vs. Control** | 0.343 | 0.648 | 0.280 | 0.343 (-0.927, 1.613) |
| **cycle2vs.1** | -0.648 | 0.362 | 3.200 | -0.648 (-1.358, 0.062) |
| **cycle3vs.1** | -0.984 | 0.425 | 5.350 | -0.984 (-1.817, -0.151)* |
| **cycle4vs.1** | -0.175 | 0.471 | 0.140 | -0.175 (-1.097, 0.747) |
| **5-HT₃ + NK-1 receptor antagonist vs.** **5-HT₃ receptor antagonist** | 0.601 | 0.350 | 2.950 | 0.601 (-0.085, 1.287) |
| **Commercial/service industry practitioner vs.** **Worker/Farmer** | -0.693 | 0.556 | 1.560 | -0.693 (-1.783, 0.397) |
| **Staff of public institutions/Civil servant vs.** **Worker/Farmer** | -0.041 | 0.501 | 0.010 | -0.041 (-1.023, 0.941) |
| **Freelancer vs.** **Worker/Farmer** | -0.792 | 0.556 | 2.030 | -0.792 (-1.882, 0.298) |
| **Others vs.** **Worker/Farmer** | 0.641 | 0.643 | 0.990 | 0.641 (-0.620, 1.902) |
| **Acupressure*cycle2** | 0.667 | 0.516 | 1.670 | 0.667 (-0.345, 1.679) |
| **TEAS *cycle2** | -0.398 | 0.459 | 0.750 | -0.398 (-1.298, 0.502) |
| **Acupressure *cycle3** | 1.059 | 0.608 | 3.040 | 1.059 (-0.133, 2.251) |
| **TEAS *cycle3** | -0.197 | 0.536 | 0.140 | -0.197 (-1.247, 0.853) |
| **Acupressure *cycle4** | -0.380 | 0.628 | 0.370 | -0.380 (-1.611, 0.851) |
| **TEAS *cycle4** | -1.508 | 0.597 | 6.370 | -1.508 (-2.678, -0.338)* |

### （2）Post-event comparison

| **Number of chemotherapy cycles** | **comparison** | **β (95% CI)** |
| --- | --- | --- |
| **cycle1** | Acupressure vs Control | 1.246 (-0.081, 2.574) |
|  | TEAS vs Control | -0.343 (-1.897, 1.211) |
|  | TEAS vs Acupressure | 1.590(0.025, 3.155)* |
| **cycle2** | Acupressure vs Control | -0.579 (-1.645, 0.487) |
|  | TEAS vs Control | -0.055 (-1.453, 1.343) |
|  | TEAS vs Acupressure | 0.524 (-0.768, 1.816) |
| **cycle3** | Acupressure vs Control | -0.187 (-1.390, 1.016) |
|  | TEAS vs Control | 0.146 (-1.275, 1.567) |
|  | TEAS vs Acupressure | 0.333 (-1.089, 1.755) |
| **cycle4** | Acupressure vs Control | -1.626 (-2.750, -0.502)* |
|  | TEAS vs Control | -1.165 (-2.521, 0.190) |
|  | TEAS vs Acupressure | 0.461 (-0.720, 1.642) |

## 5. Depression

### （1）The results of generalized estimating equation analysis

| **Variable** | **β (Estimate)** | **Std. Error** | **Wald χ²** | **β (95% CI)** |
| --- | --- | --- | --- | --- |
| **Acupressure vs. Control** | -0.843 | 0.474 | 3.170 | -0.843 (-1.772, 0.086) |
| **TEAS vs. Control** | 0.594 | 0.572 | 1.080 | 0.594 (-0.527, 1.715) |
| **cycle2vs.1** | -0.046 | 0.356 | 0.020 | -0.046 (-0.744, 0.652) |
| **cycle3vs.1** | -0.175 | 0.370 | 0.220 | -0.175 (-0.900, 0.550) |
| **cycle4vs.1** | 0.302 | 0.394 | 0.590 | 0.302 (-0.470, 1.074) |
| **5-HT₃ + NK-1 receptor antagonist vs.** **5-HT₃ receptor antagonist** | -0.031 | 0.259 | 0.010 | -0.031 (-0.539, 0.477) |
| **Commercial/service industry practitioner vs.** **Worker/Farmer** | -0.064 | 0.516 | 0.020 | -0.064 (-1.076, 0.948) |
| **Staff of public institutions/Civil servant vs.** **Worker/Farmer** | 0.898 | 0.456 | 3.870 | 0.898 (0.005, 1.791)* |
| **Freelancer vs.** **Worker/Farmer** | 0.028 | 0.579 | 0.000 | 0.028 (-1.107, 1.163) |
| **Others vs.** **Worker/Farmer** | 0.951 | 0.587 | 2.620 | 0.951 (-0.200, 2.102) |
| **Acupressure*cycle2** | 0.376 | 0.454 | 0.680 | 0.376 (-0.514, 1.266) |
| **TEAS *cycle2** | -0.756 | 0.499 | 2.300 | -0.756 (-1.733, 0.221) |
| **Acupressure *cycle3** | 0.390 | 0.499 | 0.610 | 0.390 (-0.588, 1.368) |
| **TEAS *cycle3** | -0.367 | 0.505 | 0.530 | -0.367 (-1.357, 0.623) |
| **Acupressure *cycle4** | -0.267 | 0.535 | 0.250 | -0.267 (-1.316, 0.782) |
| **TEAS *cycle4** | -1.505 | 0.554 | 7.360 | -1.505 (-2.591, -0.419)** |

### （2）Post-event comparison

| **Number of chemotherapy cycles** | **comparison** | **β (95% CI)** |
| --- | --- | --- |
| **cycle1** | Acupressure vs Control | 0.843 (-0.293, 1.979) |
|  | TEAS vs Control | -0.594 (-1.966, 0.778) |
|  | TEAS vs Acupressure | 1.437 (0.134, 2.740)* |
| **cycle2** | Acupressure vs Control | -0.467 (-1.434, 0.500) |
|  | TEAS vs Control | -0.162 (-1.441, 1.117) |
|  | TEAS vs Acupressure | 0.305 (-0.869, 1.479) |
| **cycle3** | Acupressure vs Control | -0.453 (-1.513, 0.607) |
|  | TEAS vs Control | 0.227 (-0.953, 1.407) |
|  | TEAS vs Acupressure | 0.680 (-0.457, 1.817) |
| **cycle4** | Acupressure vs Control | -1.110 (-2.066, -0.154)* |
|  | TEAS vs Control | -0.910 (-2.007, 0.187) |
|  | TEAS vs Acupressure | 0.200 (-0.804, 1.204) |

## 6. Quality of life

### （1）The results of generalized estimating equation analysis

| **Variable** | **β (Estimate)** | **Std. Error** | **Wald χ²** | **β (95% CI)** |
| --- | --- | --- | --- | --- |
| **Acupressure vs. Control** | 0.896 | 2.378 | 0.140 | 0.896 (-3.765, 5.557) |
| **TEAS vs. Control** | 5.333 | 2.233 | 5.710 | 5.333 (0.957, 9.709)* |
| **cycle2vs.1** | -2.010 | 1.602 | 1.570 | -2.010 (-5.150, 1.130) |
| **cycle3vs.1** | -0.905 | 1.635 | 0.310 | -0.905 (-4.110, 2.300) |
| **cycle4vs.1** | -3.048 | 1.654 | 3.400 | -3.048 (-6.290, 0.194) |
| **5-HT₃ + NK-1 receptor antagonist vs.** **5-HT₃ receptor antagonist** | 1.210 | 1.311 | 0.850 | 1.210 (-1.360, 3.780) |
| **Commercial/service industry practitioner vs.** **Worker/Farmer** | 4.082 | 2.189 | 3.480 | 4.082 (-0.208, 8.372) |
| **Staff of public institutions/Civil servant vs.** **Worker/Farmer** | 0.669 | 1.945 | 0.120 | 0.669 (-3.143, 4.481) |
| **Freelancer vs.** **Worker/Farmer** | 0.477 | 2.113 | 0.050 | 0.477 (-3.664, 4.618) |
| **Others vs.** **Worker/Farmer** | -0.822 | 2.561 | 0.100 | -0.822 (-5.842, 4.198) |
| **Acupressure*cycle2** | 3.737 | 2.067 | 3.270 | 3.737 (-0.314, 7.788) |
| **TEAS *cycle2** | 4.826 | 2.010 | 5.760 | 4.826 (0.886, 8.766)* |
| **Acupressure *cycle3** | 3.350 | 2.395 | 1.960 | 3.350 (-1.344, 8.044) |
| **TEAS *cycle3** | 4.820 | 2.027 | 5.660 | 4.820 (0.847, 8.793)* |
| **Acupressure *cycle4** | 6.824 | 2.387 | 8.180 | 6.824 (2.146, 11.502)** |
| **TEAS *cycle4** | 8.513 | 2.116 | 16.180 | 8.513 (4.366, 12.660)*** |

### （2）Post-event comparison

| **Number of chemotherapy cycles** | **comparison** | **β (95% CI)** |
| --- | --- | --- |
| **cycle1** | Acupressure vs Control | 0.896 (-5.557, 3.765) |
|  | TEAS vs Control | 5.333(0.957, 9.709 )* |
|  | TEAS vs Acupressure | 4.437 (0.691, 9.565) |
| **cycle2** | Acupressure vs Control | 4.634 (-0.503, 9.771) |
|  | TEAS vs Control | 10.159 (4.668, 15.650)*** |
|  | TEAS vs Acupressure | 5.525 (0.279, 10.771)* |
| **cycle3** | Acupressure vs Control | 4.246 (-0.596, 9.088) |
|  | TEAS vs Control | 10.150 (5.461, 14.839)*** |
|  | TEAS vs Acupressure | 5.904 (1.033, 10.775)* |
| **cycle4** | Acupressure vs Control | 7.720 (3.081, 12.359)*** |
|  | TEAS vs Control | 13.846 (8.888, 18.804)*** |
|  | TEAS vs Acupressure | 6.126 (1.890, 10.362)** |
